# Axiology and dynamics of contemporary research groups: a systematic review and hermeneutic meta-analysis of knowledge, values, and social elements

**DOI:** 10.3389/frma.2025.1525587

**Published:** 2025-08-07

**Authors:** Edgar O. Ayala, Myriam M. Altamirano-Bustamante, Adalberto de Hoyos-Bermea

**Affiliations:** ^1^Cross-Functional Group in Clinical Ethics, XXI Century National Medical Center, Mexican Social Security Institute, Ciudad de México, Mexico; ^2^Centro de Investigaciones Económicas, Administrativas y Sociales, Instituto Politécnico Nacional, Ciudad de México, Mexico; ^3^Metabolic Diseases Research Unit, XXI Century National Medical Center, Mexican Social Security Institute, Ciudad de México, Mexico

**Keywords:** scientific practices, system values, scientific communities, research groups, social dynamics, research dynamics

## Abstract

**Introduction:**

This research explores the practices of current research groups, where multidisciplinary groups stand out for their relevance in developing science. The objective is to comprehensively explore the practices and values of research groups through a three phase study.

**Methods:**

In the first phase a systematic review was performed, in the second phase, a hermeneutic analysis focused on values, knowledge and the social elements to describe and delve into the development of scientific practices. The third phase, a discussion of these practices and interactions was proposed.

**Results:**

The most relevant findings are the specific attitudes valued in social interactions, such as the relationship between cooperation and solidarity, economic values like the link between benefit and profitability, and research values like the connection between inclusion and coherence.

**Discussion:**

These relationships help define the value networks that shape interactions, practices and relationships within scientific groups. This describes the dynamic of contemporary science to answer today's challenges.

## 1 Introduction

Research groups, understood as voluntary associations of researchers collaborating on one or several research topics to generate new knowledge, are immersed in dynamics, such as traditions, behavioral practices, attitudes and ways of acting in different domains of knowledge, are of great relevance for scientific communities. Practice is understood as a dynamic system that includes at least one of the following elements: (a) a set of agents with common capabilities and purposes, (b) an environment in which the practice develops, (c) a set of objects that are also part of the medium, (d) a set of actions that are planned and executed based on many elements, including beliefs and theories that guide the actions of the agents (Olivé Morett, 2011). This is due to them setting the pace, the growth rate of the knowledge, values and social development of these communities—them being scientific communities, multidisciplinary groups or cross-functional teams, among others—that work to solve specific problems in local contexts. In a knowledge society, the involvement of society, users, specific communities or epistemological communities in research projects is a clear example of how knowledge production must be adapted to the contexts in which the products or results of such research are intended to be applied, since the dynamics of science are imbued with social, political, economic, and religious values that play a fundamental role in these specific contexts, necessary for solving problems and ensuring they are best managed. Therefore, in this phase where the diversity of actors in the research groups is broad, mediation, decision making, and negotiation processes must be taken into account, so that all those involved in the work of science, experts or users, contribute their own ideas to solve, communicate and transfer their knowledge of the specific problems they face.

For instance, there are several proposals (Braun and Schubert, 2003; Bainbridge et al., 2010; Andersen, 2016; Ankeny and Leonelli, 2016) that explore the dynamics within multidisciplinary groups. Other researchers (Hoekstra et al., 2018; Tittlemier et al., 2022) focus specifically on the internal interactions within research groups. The preceding favors the identification of models, proposals or frameworks that ease the collaboration process among the members of these types of groups or epistemic communities to make their activities more efficient. The community of practice (COP) or the science of team science (SciTS) are some of the current proposals headed for the study of this kind of topic. Multiple forms of teamwork are known. For example, interdisciplinary team, multidisciplinary groups or the cross-functional groups, which are also formed by professionals from different domains of knowledge who provide ideas and methodology specifics to their domains to solve a shared problem (Méndez Jiménez, 2014).

These groups embody the diversity of today's scientific communities, where science influences various social, political, and value-based factors to achieve its goals. Contemporary science promotes the enrichment of both members from diverse knowledge domains and the theoretical and methodological foundations necessary for comprehensive research. The works that analyze these types of groups are essential in fields such as educational and governmental institutions, industries, enterprises, companies, etc. since they are topics of great importance for those scenarios where comprehensive research, meaning, where a cross-functional approach is attempted. Therefore, in order to understand the topic of research groups and the dynamics among their members, it is vital to answer questions, such as “what current proposals or models of research groups exist?” or “what kind of practices can be found among the members of the groups in these proposals?”. In this systematic review, the aim is to identify qualitative and mixed studies that explore the most common practices that are being applied in current science within this groups. Subsequently, the aim is also to provide a meta-analysis that allows us to describe, classify and study in more detail the construction of a roadmap that can be walked through to improve and evolve these types of practices in these research groups.

A way of finding the practices and axiological patterns of these types of groups—the aim of this research—is to understand their value system that is at the core of their decision-making. This means that a key element of the scientific practice is its axiological structure, understood as the set of rules, regulations or principles shared by the members of these groups (Olivé Morett, 2011). Nowadays, science is practiced with different value systems to those of traditional science, meaning the value system has changed and this affects or modifies the way in which this type of scientific practice is being done (Echeverría, 2003), which, for the most part, is done within these diverse research groups. While the definition of axiological structure is broad and encompasses a wide range of elements (norms, rules, or principles), these are precisely the elements that each member of a group brings with them within the group, such as beliefs and particular ways of acting. Many of these elements will be shared and others will not, but those that are shared, such as norms or beliefs, are what determine the shape of this axiological structure, originating from institutional regulations or particular ways of acting. This is why values, rules, norms, etc., make up these “social elements” that influence the practice of scientific work. The diversity and complexity of values involved in the production of knowledge within these groups determines the structure of that value system. Therefore, this structure is more complex than just a set of values. This is why research attempts to uncover this complex system and network of values that is configured within research groups and that determines practices and, therefore, decision-making (Olivé Morett, 2011; Echeverría, 2003).

The most prominent findings of this research relate to the dominant value systems within these groups. The prevalent dynamics among members are shaped by interactions based on social values, such as cooperation and solidarity; research values, like inclusion and coherence; and economic values, such as benefit and profitability. In contrast, dynamics involving aesthetic, ecological, or religious values are less commonly observed.

In general, the most common practices were found to be among the following 10 most repeated value pairs: cooperation-inclusion, benefit-inclusion, cooperation-benefit, continuous learning-cooperation, solidarity, continuous learning-inclusion, benefit-meetings, conversations-meetings and applicability-cooperation.

The importance of studying scientific communities—particularly those that have recently emerged and combine all groups, like the cross-functional groups—is to highlight their practice, since their dynamics benefit the transmission, generation and dissemination of new knowledge and its transfer to the society's most productive sectors. It is very important to have sufficient collaboration between institutions and organizations, different levels of basic and applied research, and the critical mass in a cross-functional group, that allow the organization of resources and knowledge. One of the areas of science that has benefited the most from the work (intervention) of other sciences is health, since its research, in many cases, requires broader approaches, such as analyzing the social determinants of access to health, the different types of treatments according to geographic regions, etc.; also studies on mental health to understand its origins due to work pressures, stress, etc. Also in health, there are several emerging issues that must be considered and analyzed in detail, as with issues of ethics, since current issues such as transhumanism do not have a well-founded ethical problem posed by the physical improvements that could be made to specific sectors of society. This is an example of how, with the help of different sciences and best practices, relevant problems in science can be addressed more efficiently.

The data presented may contribute to the development of a model that allows for the establishment of a set of practices and dynamics, such as those presented above, that can make more efficient the work carried out in the area of knowledge generation, especially in these research groups. This is in light of the fact that future research will present more conclusive results on a model of the practices and dynamics of these diverse research groups. Covering the epistemic trajectories and the cross-functional value systems will allow the generation and design of humanized, comprehensive and evolved practice models to achieve a better contemporary science practice that is a source of wealth and social wellbeing.

## 2 Materials and methods

The present research was conducted in accordance with the characteristics of a systematic review, following the PRISMA (Preferred Reporting Items for Systematic Review and Meta-Analysis) and PIO (Participants, Intervention and Outcomes) approach, emphasizing a detailed description of the steps taken. The search for information was accomplished by taking into account the background of four of the most important databases: PubMed is one of the most recognized databases for biomedical and public health research; BIREME is a key database for health studies, with access to scientific literature in Spanish and Portuguese, expanding the diversity of sources and perspectives in research; Web of Science has broad multidisciplinary coverage, providing a comprehensive view of the evolution of knowledge in various areas; and Philosopher's Index is a crucial database for studies that address epistemological and axiological aspects of science, in a time span between the years 2010 and 2023.

**Figure 1:**
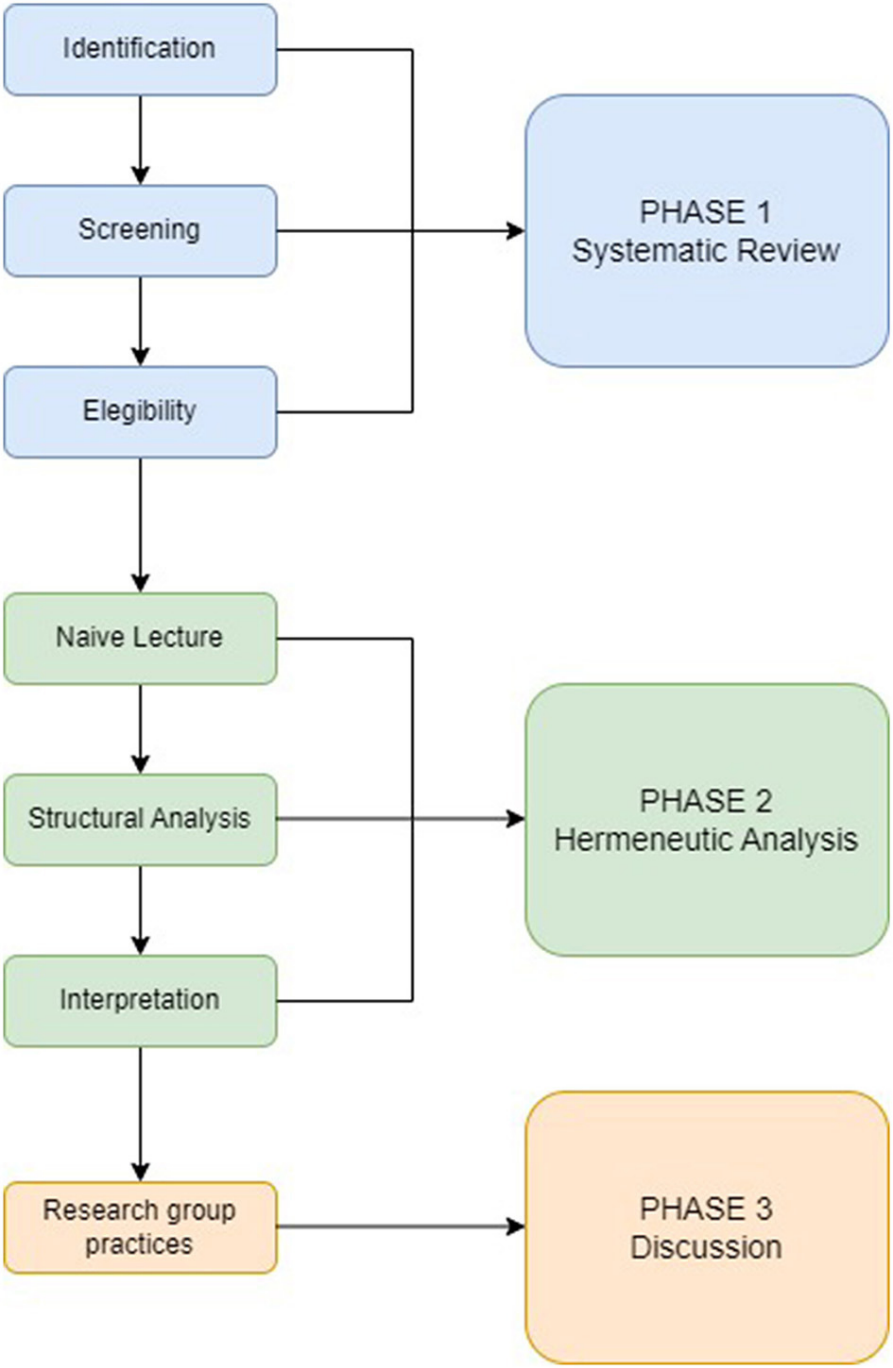
Research phases. The figure shows the three stages of this research and their respective processes.

As already mentioned, this study is divided into three main phases, Figure 1 shows its structure and central elements of each phase.

### 2.1 Phase one. PIO and PRISMA strategy

The PICO and PRISMA approaches were used to answer the research question: What are the research practices and axiological foundations that are being applied in technoscience and contemporary science within multi, inter, transdisciplinary and cross-functional groups? to formulate a clear research question aligned with the study's objectives. Carrying out the systematic review from these two perspectives enhances the results; first, because with the use of PIO we start from a well-defined research question that dissects each of its related terms; second, with the use of PRISMA, the resulting papers are found, evaluated and compared to find their similarities, differences, methods and tools used. Therefore, the use of both perspectives in this review complements the analysis of the documents found, PIO for one part of the analysis and PRISMA for another.

The PRISMA review framework has been used to carry out social research in several works (Serrano-Zamago and Altamirano-Bustamante, 2021; Vedrenne-Gutiérrez et al., 2021), and it is also considered pertinent to use the same system given its rigor and strength for the construction of a systematic review; Therefore, this framework is used in this research along with PICO. This framework is widely used in systematic reviews to structure research questions clearly and precisely, facilitating the search and selection of relevant studies. The PICO system uses four elements as a guide for the research: participants (P), intervention (I), comparison (C), and outcomes (O). Given that the element “comparison” is not relevant in this research, the PIO variant is used, where the focus is on the population of interest or participants, on the intervention within that population and on the outcomes or effects of the intervention on the participants, in this sense PIO is used when the objective is to explore the impact of an intervention without contrasting it with an alternative. This type of framework has been successfully used in other research and that is why it has been chosen to be used as reference (Juárez-Villegas et al., 2021; Monroy-Fraustro et al., 2021).

#### 2.1.1 PIO strategy

In this case, the elements that include each one of the parts of the PIO system were searched in relation to the central topic discussed above, that is, identify studies that explore most common practices models that are being applied in current techno and contemporary science within cross-functional groups. Thus, the PIO elements found in this question are the following: (I) *What are the most common practices models* (O) *that are being applied* (P) *in technoscience and contemporary science within multi, inter, transdisciplinary and cross-functional groups?* First, Participants (P): technoscience and contemporary science within multi, inter, transdisciplinary and cross-functional groups; Second, Intervention (I): the most common practices models; Third, Outcomes (O): that are being applied. In this manner, the keywords for each element of PIO are:

P = Scientific communities, techno scientific group, technoscientific group, techno scientific communities, technoscientific communities, epistemic communities, practical endorsement, multidisciplinary group, multidisciplinary communities, transdisciplinary group, transdisciplinary communities, interdisciplinary group, interdisciplinary communities, cross-functional group, cross-functional communities, biomedical scientific communities, biomedical scientific society, biomedical scientific group, research group and practice.
I = Epistemic models, scientific models, scientific networks, scientific approach, theoretical model, practical scientific model, techno scientific model, technoscientific model, multidisciplinary model, interdisciplinary model, transdisciplinary model, cross-functional model, pluralistic model and social model.
O = Productivity, applicability, sociology, axiology, knowledge, communication, networks, skills, cohesion, education, scientific practice and scientific conception.

To search for P elements, the main actors in current scientific work were identified. Terms such as “Biomedical Scientific Society” and “Biomedical Scientific Group” help identify specific practices within this research, key to technoscience. Terms such as “Multidisciplinary Group,” “Transdisciplinary Group,” “Interdisciplinary Group,” “Cross-Functional Group,” and “Cross-Functional Communities” seek research that explores the interaction between groups from different disciplines in the generation of knowledge. Using these terms in the search broadens the spectrum, ensuring that relevant studies on the most common practices in technoscience and contemporary science are identified within these research groups.

#### 2.1.2 Search strategy following PICO and PRISMA approach

These are all the MeSH keywords and terms used in the search in each one of the databases mentioned above. When searching for each of these terms and the subsequent combinations between terms and parts or items of PIO, the Boolean operators “OR” and “AND” were, respectively, used. This exercise is described below:

To find the results of each MeSH term of the three items of the PIO system, they were searched individually one by one in every database, following the own nomenclature of each database, either the word alone or in quotation if it is more than one word (education o “scientific communities”).
To combine all the terms of each item, the Boolean “OR” was used between each term: (scientific communities) OR (technoscientific group) OR …
To combine all the terms with another term, the Boolean “AND” was used between each term, in this way those papers that did not have those terms relevant to the research were eliminated: ((scientific communities) OR (technoscientific group) OR …) AND (practice).
To combine all the terms of an item with another item, the Boolean “AND” was used between each term or the result of the whole combination of an item: ((scientific communities) OR (techno scientific group) OR …) AND ((epistemic models) OR (scientific models) OR …).

#### 2.1.3 Decision tree description of each of the databases

##### 2.1.3.1 PubMed

When searching in the PubMed database, all the MeSH terms of the item “P” described above were firstly searched, that way, the results obtained were 28,964 as the total result of “P.” Subsequently, all the “I” terms were searched and added together, obtaining 67,324 results. The same was done with the “O” terms, obtaining a total of 7,867,948. Finally, the combination of “P” total with “I” total was made using the Boolean “AND,” obtaining 2,801 as the result, and this result was combined in the same way with that of “O” total to arrive at 2,144 as the result of works found in this database.

##### 2.1.3.2 BIREME

The search in the BIREME database was conduct in a similar way to the previous one, obtaining a result of 6,396 as the total of item “P.” In the same way, all the MeSH terms of item “I” were searched and then added using the Boolean “OR” to obtain a result of 470,772. The same was done with the terms of item “O,” whose total result with the Boolean “OR” was 3,120,508. The result of item “I” was combined with that of item “P” with the Boolean “AND” and the result was 1,374. Finally, that result was combined with that of item “O,” reaching a total of 1,060 works in the research of this database.

##### 2.1.3.3 Web of science

The next search was conducted in the Web of Science database. The same way as the previous ones, a search was conducted for each of the MeSH terms of item “P” described above, obtaining a total result of 3,313 for the item “P.” The next step of the search focused on all the terms in item “I” to then make the total conjunction with the Boolean “OR,” obtaining 961,737 as the result. This result was combined with the total of item “P” using the Boolean “AND” to obtain 1,032. Then all the terms in item “OR” were searched and combined using the Boolean “OR” to obtain 6,001,578. Finally, this result was combined using the Boolean “AND” with that of the combination of the items “P” with “I,” obtaining as the result of the research on this database 864 works.

##### 2.1.3.4 Philosopher's index

The last database where the search and combination of MeSH terms was conducted was Philosopher's Index. Following the same methodology, the item “P” was the first, searching each term individually and then combining it with the Boolean “OR,” to obtain a total of 167 for item “P.” Then, the “I” terms were searched and combined using the Boolean “OR” to obtain a total of item “I” of 3,101. Later, they were combined with the results of “P” using the Boolean “AND,” which obtained a result of 10. The terms of the last item “O” were searched individually, to be then combined in a general way using the Boolean “OR” to obtain a total of 94,120. Finally, this result was combined with the result of the combination of the item “P” with the “I” to obtain a result of 10 works in this database.

#### 2.1.4 PRISMA flowchart

After the search of the material, the works were filtered to obtain the most relevant ones for the objectives proposed at the beginning of the research. In this part of the sieving, the Mendeley program was used as a tool to obtain bibliographic data and also as a computer and organizer of all the works. The first thing that was done was to discard those works that did not have enough relevant data, such as the title, authors, abstract or the journal that published it.

The total results were obtained from the different databases: the highest number of results was found in PubMed, with a total of 2,030 papers; followed by BIREME, where a total of 1,050 papers were found; next comes Web of Science, where a total of 685 papers were obtained; and finally, the database where the fewest works were obtained in the search was Philosopher's Index, with a total of 10 works found. The total results of all the databases were 4,078 works, and 5 from other sources.

The second filtration was to dismiss articles with a double reference, around 519. Then, those that were not written in English or Spanish, review works and conference proceedings, and those whose topics were not relevant to the research topic, and a keyword search in each of the works was also made. The keywords used were “scientific community” and “scientific communities,” “model,” “practice,” and “science.” Once the filtration was done, the number of dismissed works was 3,870, leaving a selection of just 208 works.

A further and more detailed filtration was made focused on the quality criteria, based on a detailed and rigorous reading of the titles and abstracts of the works, leaving a selection of just 45 works. Then, a final reading dismissed 13, leaving a final 32 selected works.

#### 2.1.5 PRISMA assessment of the quality of the included studies

To find a way to establish a detailed assessment of the articles found, some parameters were used to evaluate them, among them are the objectives, the research question, the methodology, definition of relevant terms and the results.

Regarding the objectives, they have to be clear from the beginning, express what you want to look for in a detailed and formal way; as for the research question, it must also be shown clearly and formally, but if it is expressed as a relevant topic or a narrative expression, it is difficult to make sense of it and can cause confusion; on the other hand, the methodology must also be expressed clearly, if possible from the abstract or in the specific sections for that task, and be congruent with what you want to find, that is, be appropriate for the study; regarding the definition of relevant terms, it is important to expose the framework in which the terms are used to understand, from the beginning, what is meant by those fundamental concepts of the study; finally, the results must be in accordance with the objectives. If topics that were not reviewed from the objectives are explained in this section, it is thought that there is no congruence in the study.

Each parameter that evaluates the quality of the study has a maximum value of twenty, if what is analyzed is satisfactorily fulfilled, but if it is not expressed clearly or does not comply with what is proposed, the minimum value can reach zero. By adding all the parameters, the final score of the study was obtained, which could reach 100 or a value below that.

In general, the most of studies (24) were above the value of 80, while only eight had a lower value, highlighting that only one had an evaluation of 30, which did not represent a problem for including it in the meta-analysis, the relevant to it was the topic discussed in it. Most studies (7) had an evaluation of 90, then came some (5) with 85, others (4) with an evaluation of 95, and only three studies were found with an evaluation of 100. For more information, the evaluation table is attached as Supplementary material that refers to the individual evaluation of each article studied.

### 2.2 Phase two. Hermeneutic meta-analysis of knowledge, values, and social elements

Meta-analysis is an approach used in research to analyze and synthesize existing knowledge, theories, or findings. The term “epistemic” refers to the examination of knowledge or understanding by evaluating the underlying assumptions, methodologies, and theoretical frameworks of scientific practices. This approach aims to provide a deeper understanding of the current state of knowledge within the research practices, highlighting its strengths, limitations, and areas for further investigation.

Codes and Code families were created according to the Echeverría (2003) proposed values system. These values and their respective codes were instrumentalized and defined by two of the authors based on their relevance during the process of diagnostic screening on the selected papers.

We considered these values to be very relevant in the analysis of scientific practices, they are useful to investigate the axiological and sociological factors that influence research. Every code has an operational definition, which was applied upon the different quotations recognized in the text: “the values codes were hunted” from the different articles as the reading progressed.

To carry out the analysis of the articles, hermeneutic approach was used, proposed by Ricoeur (Ricoeur, 1991) to achieve a good interpretation based on a detailed and deep understanding, in which there are several stages to carry out the analysis, the first is a naive reading, which offers a general perspective of the text and its contexts; simultaneously a quick check of the designated codes from Echeverria's values System was performed. The next stage is the structural analysis in which is a more precise reading, in which the most representative contents or fundamental ideas can be found and exhaustively coded; In the third stage, a reading is done that attempts to incorporate each of the elements of the previous stages, achieving an interpretation of the text that is as complete as possible.

**Figure 2:**
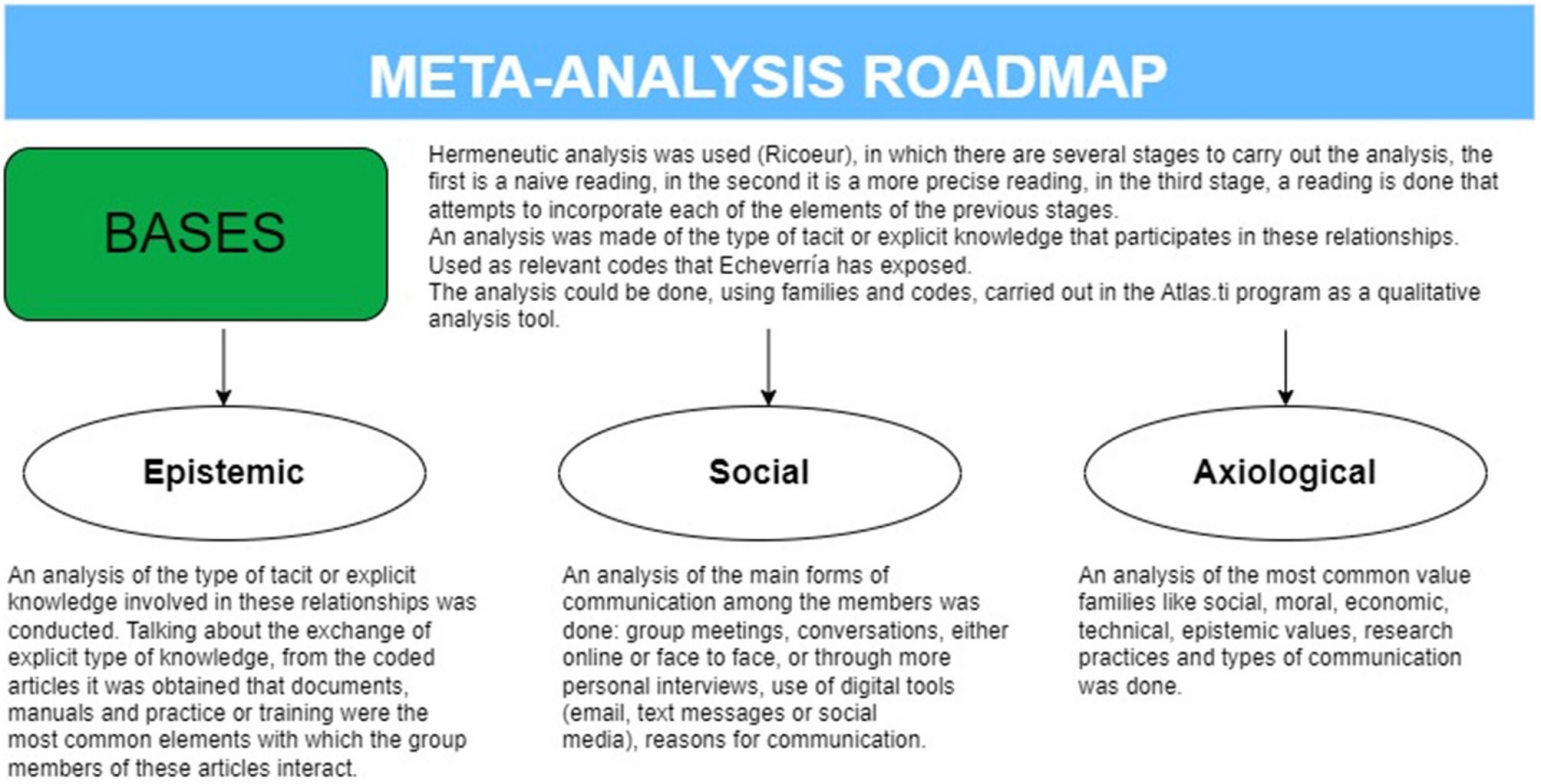
Meta-analysis roadmap. This figure details the bases of the research analysis and outlines some important results.

The first reading was conducted to identify the important topics and articles, which was followed by a second reading. To better understand the way in which the exchange of information occurs between members within the groups analyzed in the articles, an analysis was made of the type of tacit or explicit knowledge that participates in these relationships. Tacit knowledge is acquired through experience and cannot always be expressed in words. It is transmitted through gestures, attitudes, implicit norms, practices, observations, or the shared context between individuals. In other words, it involves tacit communication between individuals that requires no formal explanation. Within this process, tacit information elements (such as experiences, habits, learning styles) facilitate the exchange of information without the need for explicit documentation (Polanyi, 1966). To conduct this task, the analysis used as relevant codes or themes that Echeverría (Echeverría, 2003) has exposed in his axiological study that is present in the work of technoscience. In this way, the analysis could be done, using Echeverría's families and codes, carried out in the Atlas.ti program as a qualitative analysis tool. See Figure 2.

## 3 Results

### 3.1 Phase one. Overview of the practices of research groups

**Figure 3:**
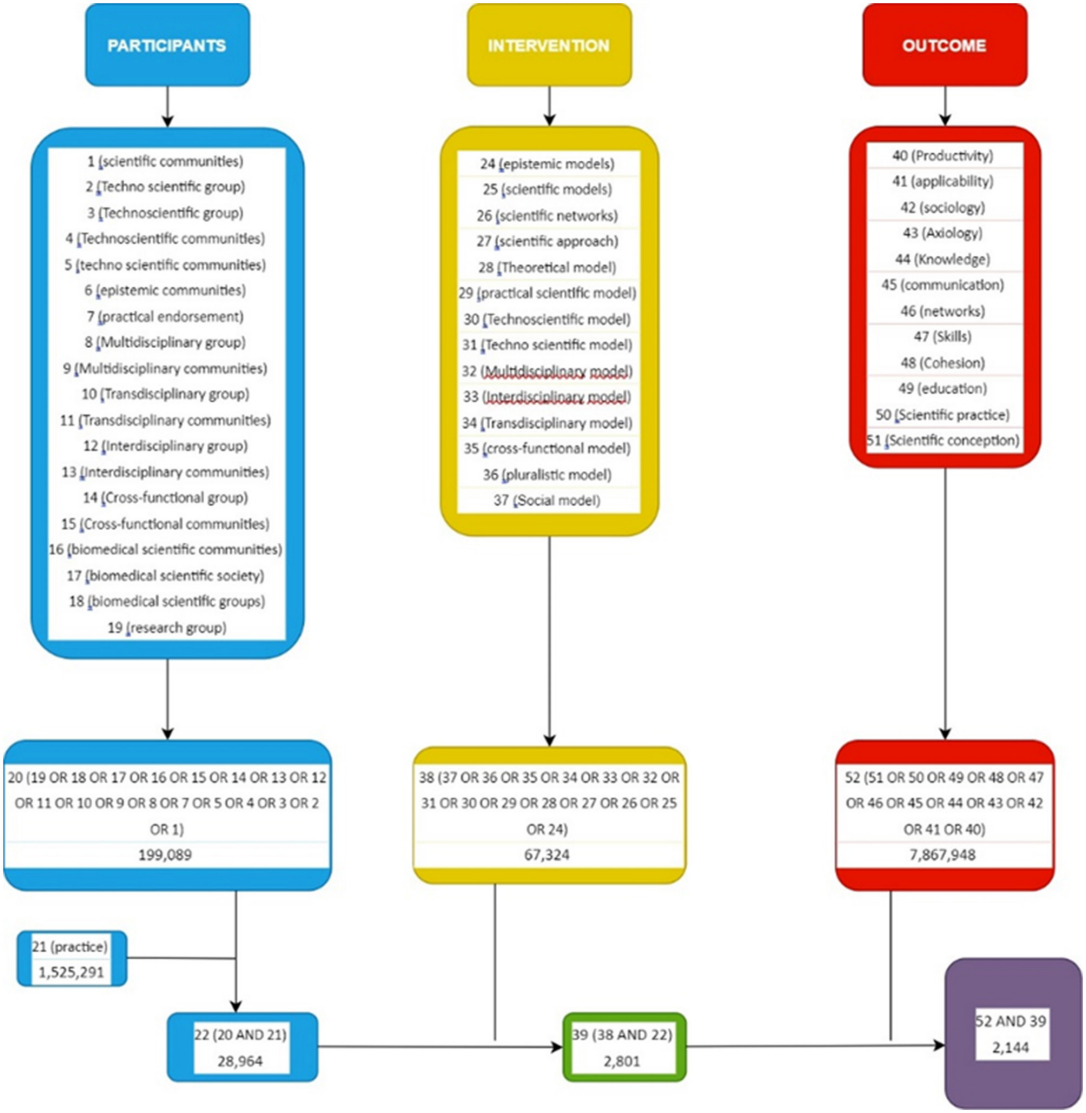
Decision tree process. The figure shows the search process in each of the selected databases, detailing the search terms separately and subsequently summing all the PIO components.

The search strategy was the PIO approach, this being participants, intervention and outcomes. Each of these elements or items included the main concepts that need to be present in the search for practice models or diverse scientific communities dynamics contextualized in the current era of technoscience. As the subjects to be analyzed are the scientific communities or the research groups—such as cross-functional ones and their practices—the terms that are part of “P” (participants) are selected in that direction, so “scientific communities,” “technoscientific communities,” “cross-functional groups,” “research groups,” or “practices” were some of these elements. Then, for the election of the elements of “I” (intervention), concepts that reflected their ways of working were searched, so the concept “model” was the main one and it was related to other useful concepts to scientific work, such as “theoretical model,” “research model,” “social model,” “plural model,” etc. Finally, to choose the elements of “O” (outcomes), concepts related to the practices of these research groups, such as “productivity,” “skills,” “networks,” “cohesion,” etc., were used. With all these elements, the search in the selected databases was started, as shown in Figure 3.

**Figure 4:**
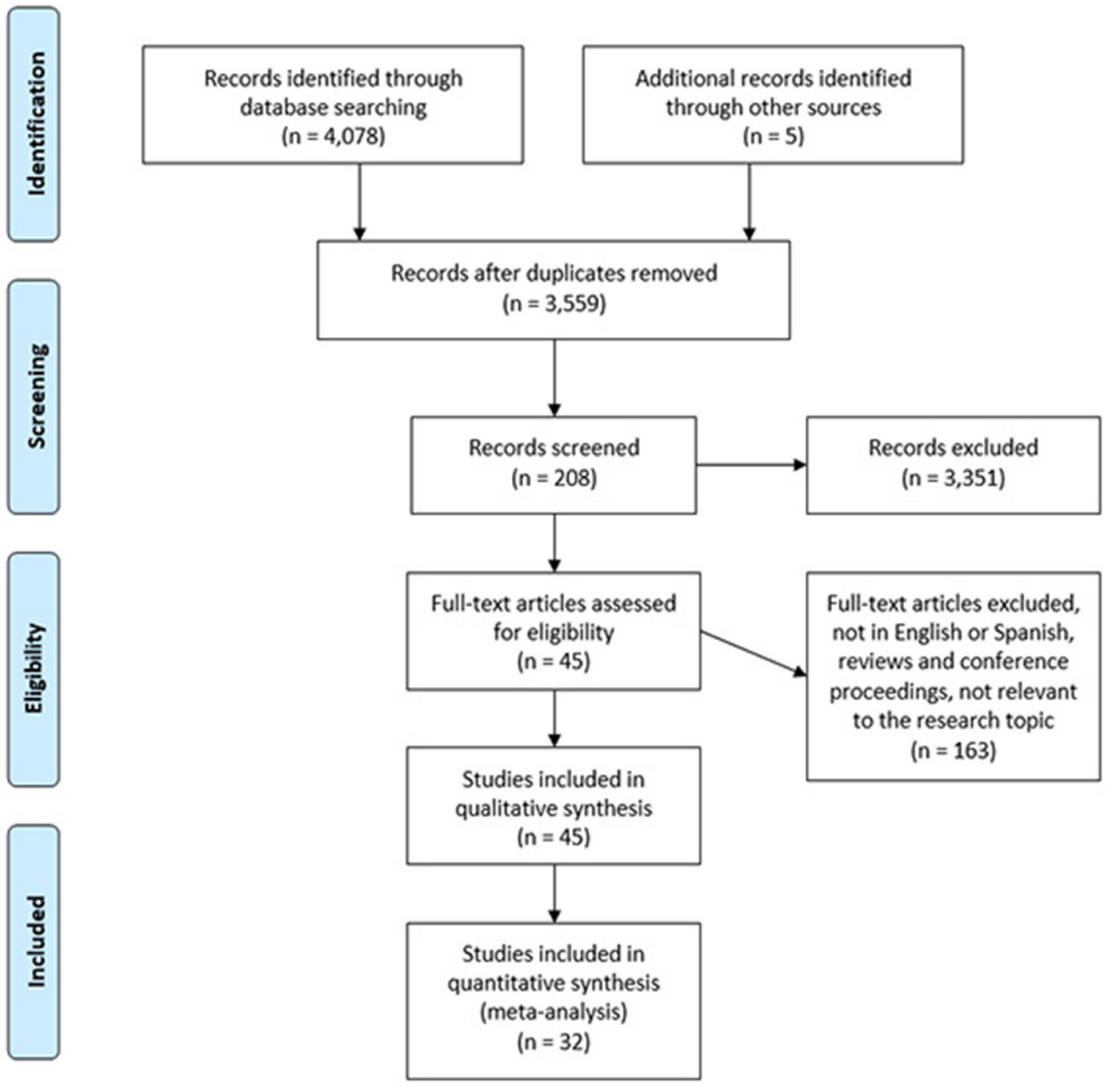
PRISMA flow diagram. The figure details the articles selection process, including the papers found from all databases, until reaching the included studies.

Four thousand documents were obtained to initiate the screening of the results using several exclusion conditions. Some of these conditions were the duplication of articles or being written in languages other than English or Spanish, or in letters or in books. Therefore, when applied to the filtration, the results were reduced to 32 final studies. Figure 4 shows the path of the filtration conditions of the documents; the final stage where only the papers to be qualitatively analyzed in the Atlas.ti program remained.

Subsequently, the review of the data of these selected articles began. The first feature found was that a third of them are an empiric type of study (Roa-Atkinson and Velho, 2005; Báscolo et al., 2006; Millerand and Baker, 2010; Zammar et al., 2010; Mcdonald and Patka, 2012; Woodruff, 2013; Erden et al., 2015; Lape et al., 2018; Beeker et al., 2021; Love et al., 2021; McCormack and Levites Strekalova, 2021; Sargent et al., 2022), while the other two thirds are a theoretical type of study (Fertman et al., 2005; Pahl-Wostl, 2007; Börner et al., 2010; Dlouhá et al., 2013; Bisol et al., 2014; Sung and Parboteeah, 2014; Umpleby, 2014; Green et al., 2015; Sanders and Kirby, 2015; Snyder et al., 2016; Grand et al., 2018; Salamone et al., 2018; Stevens et al., 2018; Lienert and Linkov, 2019; Hulcr et al., 2020; Weiskopf, 2020; Begerowski et al., 2021; Tolk et al., 2021; Beans et al., 2022; Tittlemier et al., 2022). This highlights the fact that, nowadays, when talking about studies of models of practice and interaction among the members of diverse scientific communities, we are mostly talking about theoretical analyses, which are more concerned with conceptual or structural elements than in a type of intervention focused on questions of interaction or processes of communication among the members of this types of groups.

Regarding the studies that do choose to conduct an empiric type of research, they mostly (90 of them) conduct case studies to research groups and their members. Only Erden et al. (2015) chooses to conduct an analysis to several research groups that develop their work within business or industries, focused on the reputation-economic benefit relationship for business, being the only document that addresses the economic benefit that certain research practices produces in business, such as the reputation gained by the value of their assets, results of the work of the scientific group in charge of the R+D.

The remaining articles using an empirical type of research are, as mentioned above, conducted with focus groups of interest, ranging from small communities of practitioners (Beeker et al., 2021) to large groups consisting of students and researchers from various branches of science and support staff (Roa-Atkinson and Velho, 2005; McCormack and Levites Strekalova, 2021). Several tools were used to collect data and it was found that the most consistent were surveys (36), interviews (27), and educational or learning workshops (27).

Regarding the articles that conducted a theoretical study, it was found that theoretical or conceptual analyses of specific models or proposals (Sanders and Kirby, 2015; Weiskopf, 2020; Beans et al., 2022) and reviews (Grand et al., 2018; Begerowski et al., 2021; Tittlemier et al., 2022) were the most used tools, each one signifying 38 of the total of documents. See Table 1 next.

**Table 1:** Analysis of selected articles.

**Author/year/country**	**Type of study**	**Type of analysis**	**Instruments used**	**Results**
Báscolo/(2006)/Argentina	Empirical	Hermeneutic phenomenological research. Longitudinal. A case study (2 work teams: research team and management team)	Scope mapping, workshops with the participation of the EI and the EG and individual interviews, documentary record and management reports	The interaction between the research team of the Juan Lazarte Health Institute and the management team of the Public Health Insurance is analyzed
Beans et al./(2022)/USA	Theoretical	Theoretical analysis	–	A shared definition of precision medicine research was developed
Beeker et al./(2021)/Germany	Empirical	Hermeneutic phenomenological research. Longitudinal. A case study	Notes and records from meetings and sessions, interpretative method of interactive interviewing	An atmosphere of mutual trust and respect within the group is crucial, and ongoing self-reflection or monitoring can be beneficial
Begerowski et al./(2021)/USA	Theoretical	Integrative review	–	Recommendations were presented to improve interventions in science teams by applying best practices from the team and group literature
Bisol/(2014)/Italy	Theoretical	Review and presentation of summaries of presentations and discussions	–	Encouraging interdisciplinary dialogue and synthesizing different perspectives was considered to lead to new and promising avenues for a more open science
Börner et al./(2010)/USA	Theoretical	Theoretical analysis	–	A multi-level, mixed-methods approach to SciTS is needed to gain targeted insight, foster high-impact practice, and guide effective policy on team science
Dlouhá/(2013)/Czech Republic-USA-Germany	Theoretical	Boock review	–	The integration of various perspectives that inform regional learning aimed at sustainable development
Erden/(2015)/Swiss	Empirical	Longitudinal research. A group of companies	Comparative analysis	The social evaluations of knowledge stocks by both the scientific and business communities affect firm performance
Fertman/2005/USA	Theoretical	Literature analysis	The WMAHEC interdisciplinary model was used for analysis	The interdisciplinary training project was analyzed for the construction of a collaborative, adaptable, and responsive program
Grand/(2018)/USA	Theoretical	Theoretical analysis	A review of the literature on best research practices was carried out	Various scientifically sound, evidence-based research practices with administrative and editorial practices are discussed
Green/(2015)/USA	Theoretical	Literature analysis	The background and context of the Project DataSphere data exchange platform are analyzed	Project Data Sphere creates an environment of research collaboration between industry sponsors, cancer researchers across institutions, the public, and other stakeholders
Hulcr/(2020)/USA	Theoretical	Literature analysis	The current situation of the research groups is compared with the advantages that the use of the Internet would provide	Contemporary communication strategies are proposed to interact with the global community of researchers
Lape/(2018)/USA	Empirical	Hermeneutic phenomenological research. Longitudinal. A case study	An author-generated survey was administered before and after the study to elicit views about the Kawa model's potential use as a team collaboration tool	Use of the Kawa model provides common ground for interprofessional discussions when making decisions about a client's care
Lienert/(2019)/USA-Swiss	Theoretical	Papers review	–	It's highlight advanced methodological approaches, often interdisciplinary, and include the perspectives of different stakeholders in the environmental decision-making process
Love et al./(2021) USA	Empirical	Hermeneutic phenomenological research. Longitudinal. A case study	Social network survey, Historical social network data, Retrospective team survey, Interviews, Participant observations	This case-based study provides valuable insights into the interactions that enhance scientific expertise to train interdisciplinary scientists
McCormack et al./(2021)/USA	Empirical	Hermeneutic phenomenological research. Study group of 32 doctoral students and 26 mentors	A training program was evaluated using various metrics to find the success of the program	CTS training supported increased self-efficacy for clinical research skills and resulted in a significant increase in the frequency of participation in interdisciplinary collaborative activities
Mcdonald/(2012)/USA	Empirical	Hermeneutic phenomenological research. Longitudinal. 17 participants: between 30 and 69 years of age	The Focus Group Guide and Participant Survey	Support is indicated for the use of ethical principles and newer models of disability to promote inclusion in research, and strategies to promote research participation
Millerand/(2010)/Canada-USA	Empirical	Hermeneutic phenomenological research. Longitudinal. Focal group	Qualitative research methods are used to build grounded theories and ethnographic techniques are used	Contributes to a deeper interdisciplinary understanding of the user, the multiple roles in systems development, and the dynamic sets of relationships
Pahl-Wostl/(2007)/Germany	Theoretical	Literature analysis	The comparative method is used to review several proposals	Arguments are provided for the role of social learning processes and the need to develop methods that combine physical and soft systems analysis approaches
Roa-Atkinson/(2005)/UK-Brazil	Empirical	Comparative study. Longitudinal. Bibliometric analysis and interviews (31 leading research groups)	Bibliometric study and semi-structured interviews	A database with acknowledgments was created to identify the different actors who take part in the process of knowledge production
Salamone/(2018)/USA	Theoretical	Theoretical analysis	Description and evaluation of the research model	Offered is a model that have found effective and that could be an option for those interested in developing productive, successful, and sustainable collaborations
Sanders/(2015)/Australia	Theoretical	Theoretical analysis	–	Specific quality assurance mechanisms are discussed that increase accountability, professional, and consumer confidence
Sargent/(2020)/USA	Empirical	Hermeneutic phenomenological research. Longitudinal. A case study	Using a de-identified survey, an internal evaluation was conducted	Institutions must have a process for evaluating team science
Snyder/(2016)/USA-UK	Theoretical	Literature analysis	–	Tips for communication and practices among Alzheimer's disease researchers are provided
Stevens/(2018)/USA-Holland	Theoretical	Theoretical analysis	A case study	A community of practice model is proposed that mainly addresses the areas of communication, practices and methods in these groups
Sung/(2014)/TW-UK	Theoretical	theoretical analysis	–	A new model of the Cops is proposed based on the CE, focused on practices
Tittlemier/(2022)/Canada	Theoretical	Scoping review	Descriptive statistics and narrative synthesis were used to report the results	The models and frameworks identified could be utilized by researchers and knowledge users to inform aspects of a health research partnership
Tolk et al./(2021)/USA-UK	Theoretical	Theoretical analysis	–	Based on an analysis of the benefits of modeling and simulation, it is proposed to apply this proposal for collaborative research in multi, inter and transdisciplinary environments
Umpleby/(2014)/USA	Theoretical	Theoretical analysis	–	It suggests strategies for developing second-order science. And it describes several methods that can be used to practice second-order science
Weiskopf/(2020)/USA	Theoretical	Theoretical analysis	–	The model of knowledge integration is one on which both bodies of knowledge come together into a single overarching
Woodruff/(2013)/USA	Empirical	Description of a longitudinal case study	Results are analyzed using quantitative analyzes	NIH and the public would be well-served by supporting clinical problem-based, multidisciplinary team science approaches
Zammar/(2010)/Brazil-Singapore-USA	Empirical	Longitudinal. Set of groups	Grounded theory, system dynamics, data triangulation and negative case analysis	Four major emerging themes were found: definitions, What if situations, metaphors and analogies and prolepsis

The table presents the classification of the articles, with important data such as the type of study, analysis, instruments, and their results.

### 3.2 Phase two. Hermeneutic analysis of knowledge, values and social elements of research group practices

#### 3.2.1 The relevance of tacit knowledge in group interaction

To understand the dynamics of information exchange within the analyzed, we examined the tacit or explicit knowledge involved in these interactions. The results of the analysis conducted in the Atlas.ti program are presented below.

Talking about the exchange of explicit type of knowledge, from the coded articles it was obtained that documents (30), manuals (23), and practice or training (23) were the most common elements with which the group members of these articles interact, as opposed to tutorials or software, which are the least frequent elements. This indicates that, even with recent technologies and the great progress made in recent years in the development of ICT, the traditional elements that have intervened as mediators of information among the professionals in these groups still prevail. The papers where this type of information exchange is most consistently represented are Hulcr et al. (2020), Beans et al. (2022), or Begerowski et al. (2021).

On the other hand, when tacit elements of knowledge exchanged are analyzed, there is an increased focus on them in the articles, given that they are represented more than twice as often as explicit ones, with experiences (29), learning (26), and values (16) being the most common. The articles where this tacit type of knowledge exchange is most discussed are Grand et al. (2018), Beeker et al. (2021), or Pahl-Wostl (2007).

#### 3.2.2 Social dynamics of research groups: encounters and habits

Regarding how the type of communication within the groups in these articles is described, it is known that the main forms of communication among the members of these groups are through group meetings (36.8%); with conversations (32.6%), either online or face to face; or through more personal interviews (12.6 %). The use of digital tools—such as email, text messages or social media—reaches a small percentage (4.2%) of the total communication exchange described in these articles. As shown above and following the same direction, the use of more recent technologies is extremely low compared to traditional means of communication exchange. Some of the papers that show these diverse ways of communication are Begerowski (Begerowski et al., 2021), Beeker (Beeker et al., 2021) or Woodruff (Woodruff, 2013).

There is little information regarding the motives or reasons for the exchange of communication among the members of the research groups described in the articles. This is because almost all the studies focus on academic reasons or motives related to the information about the problem to be solved. Only two papers (Sung and Parboteeah, 2014; Beeker et al., 2021) address social or cultural reasons among the motives for communication, these being related to a more personal interaction between the members of these groups.

**Figure 5:**
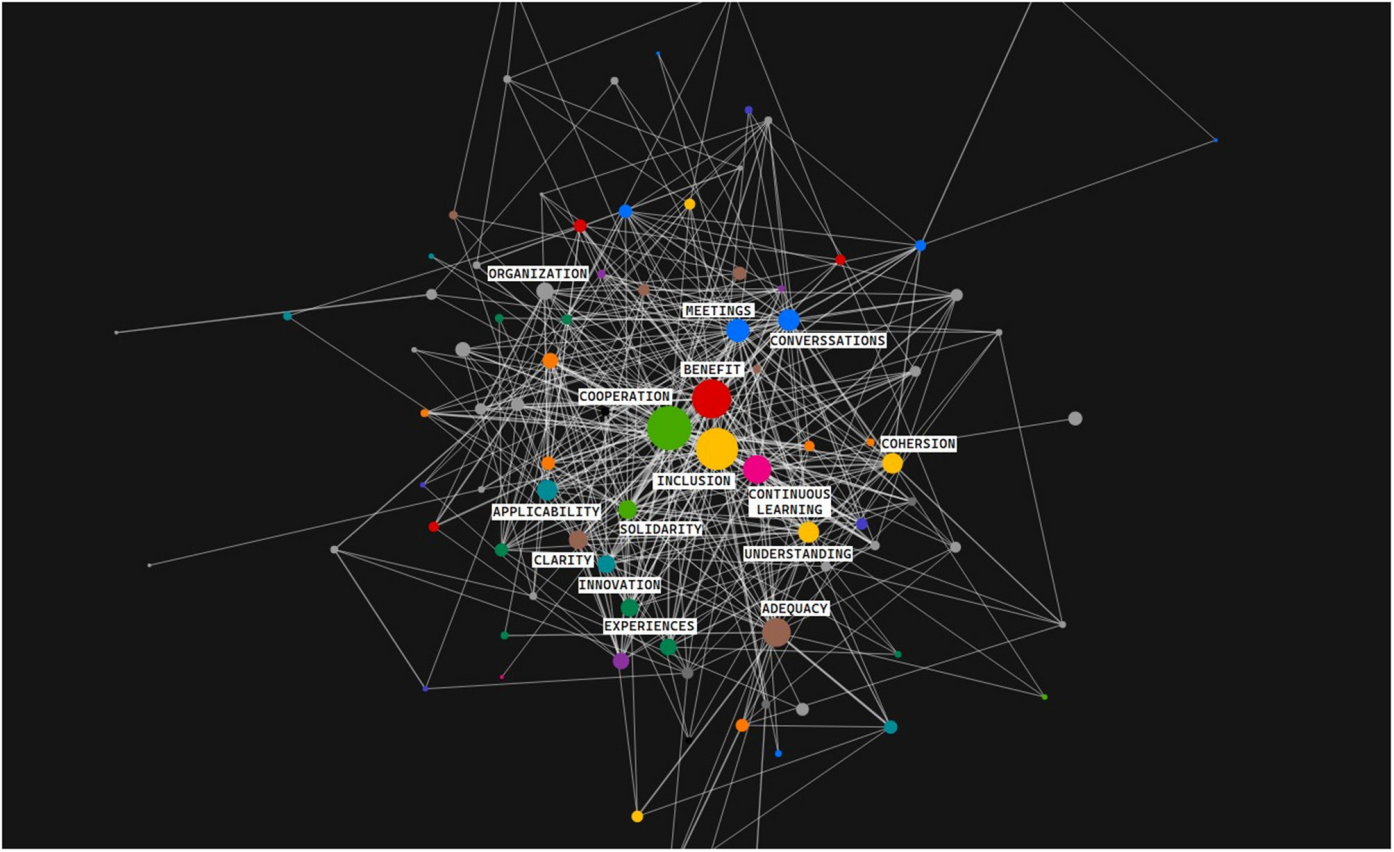
Connections and centrality of value. This figure shows the most common codes from the analysis and their relationship with others, generated with Atlas.ti.

**Figure 6:**
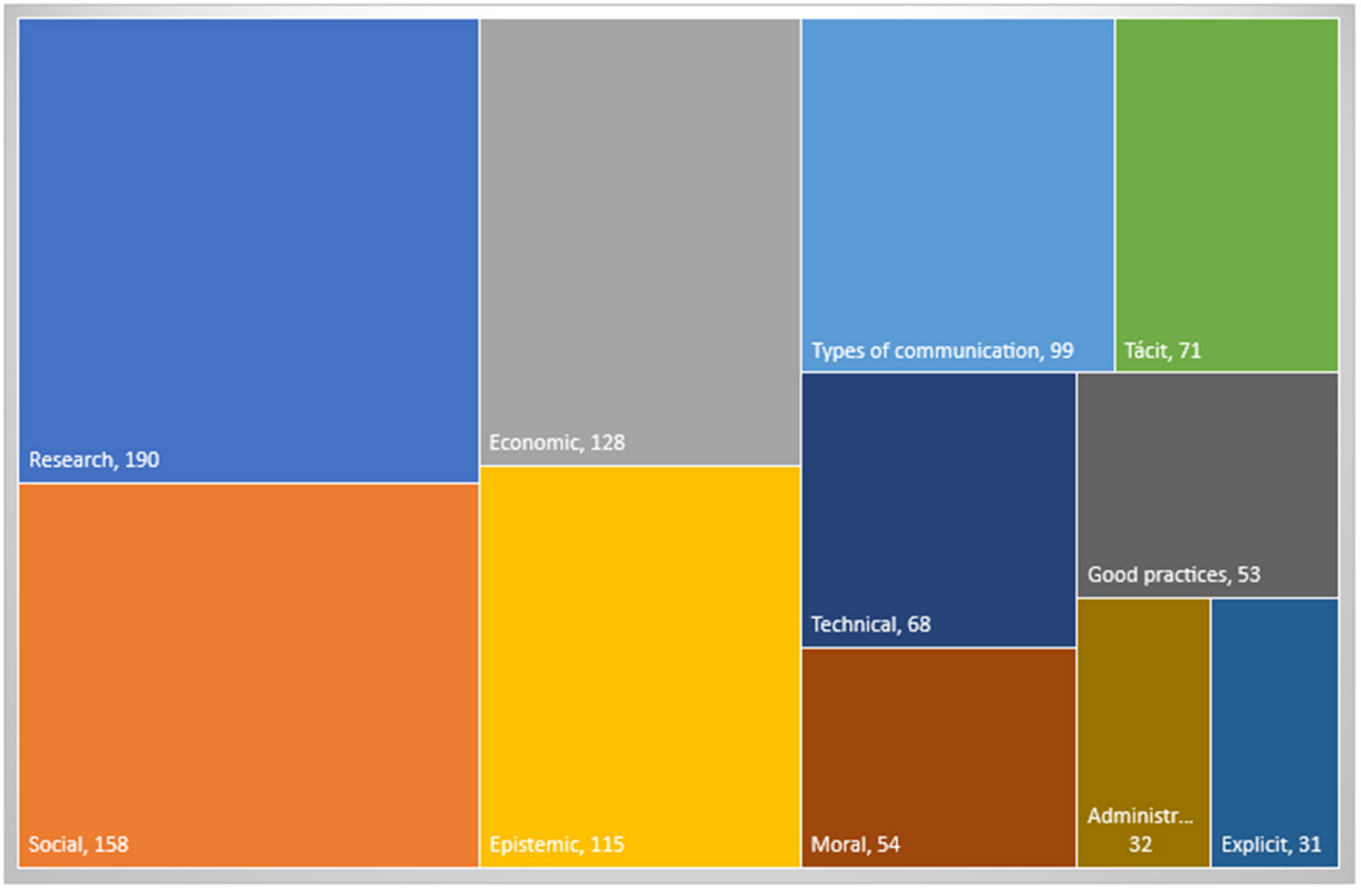
Values families. The figure presents the most common families of values and practices that resulted from the document analysis, generated with Atlas.ti.

**Figure 7:**
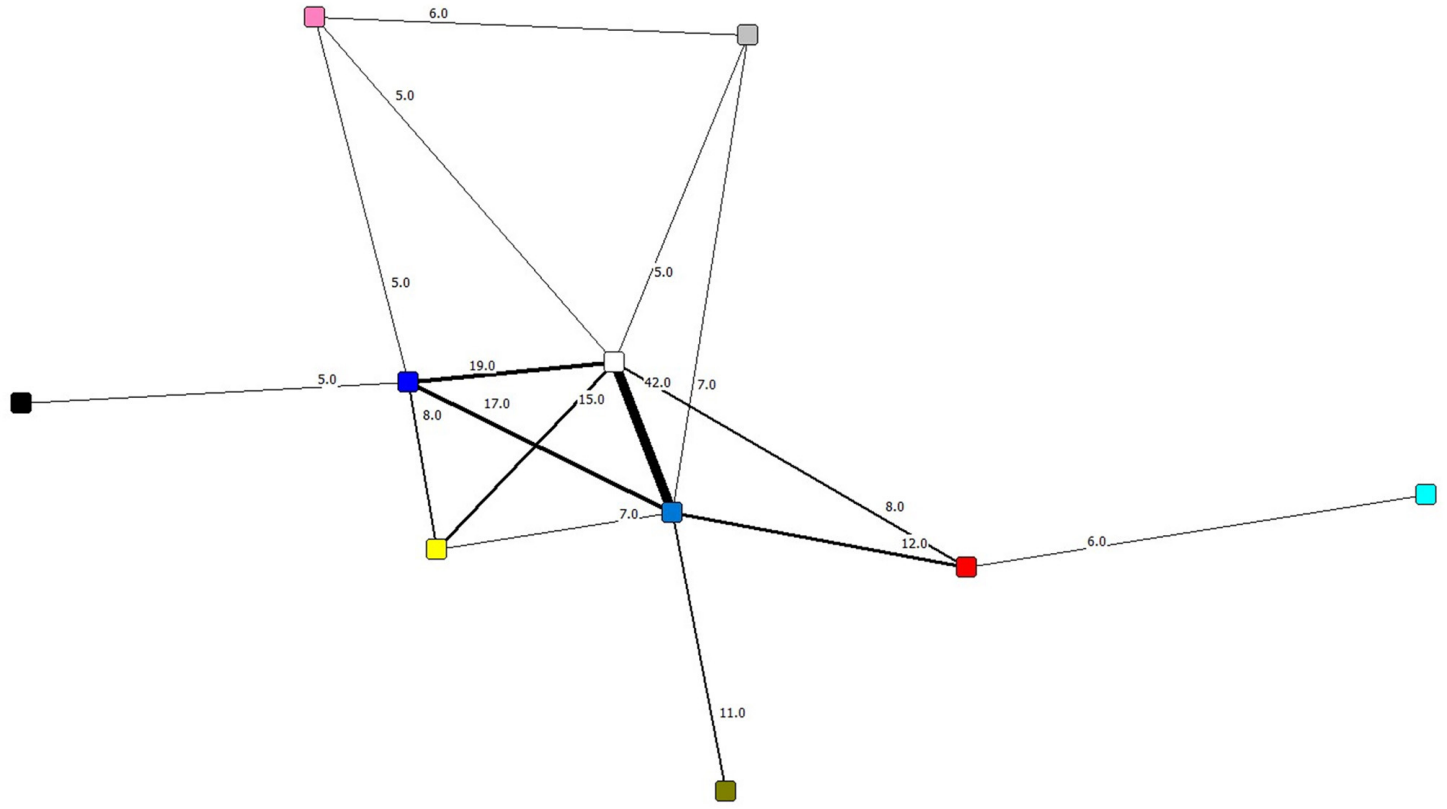
Relations between families. This figure shows the most representative families of values and practices and their centrality, generated with Ucinet. The colors of the nodes are explained below. Families color: Yellow, Types of communication; Blue, Social values; White, Research practices; Brown, Moral values; Gray, Technical values; Navy, Economic values; Black, Explicit knowledge; Red, Good research practices; Pink, Epistemic values; Turquoise, Tacit knowledge. The numerical value between one family and another is the frequency with which they were related.

**Figure 8:**
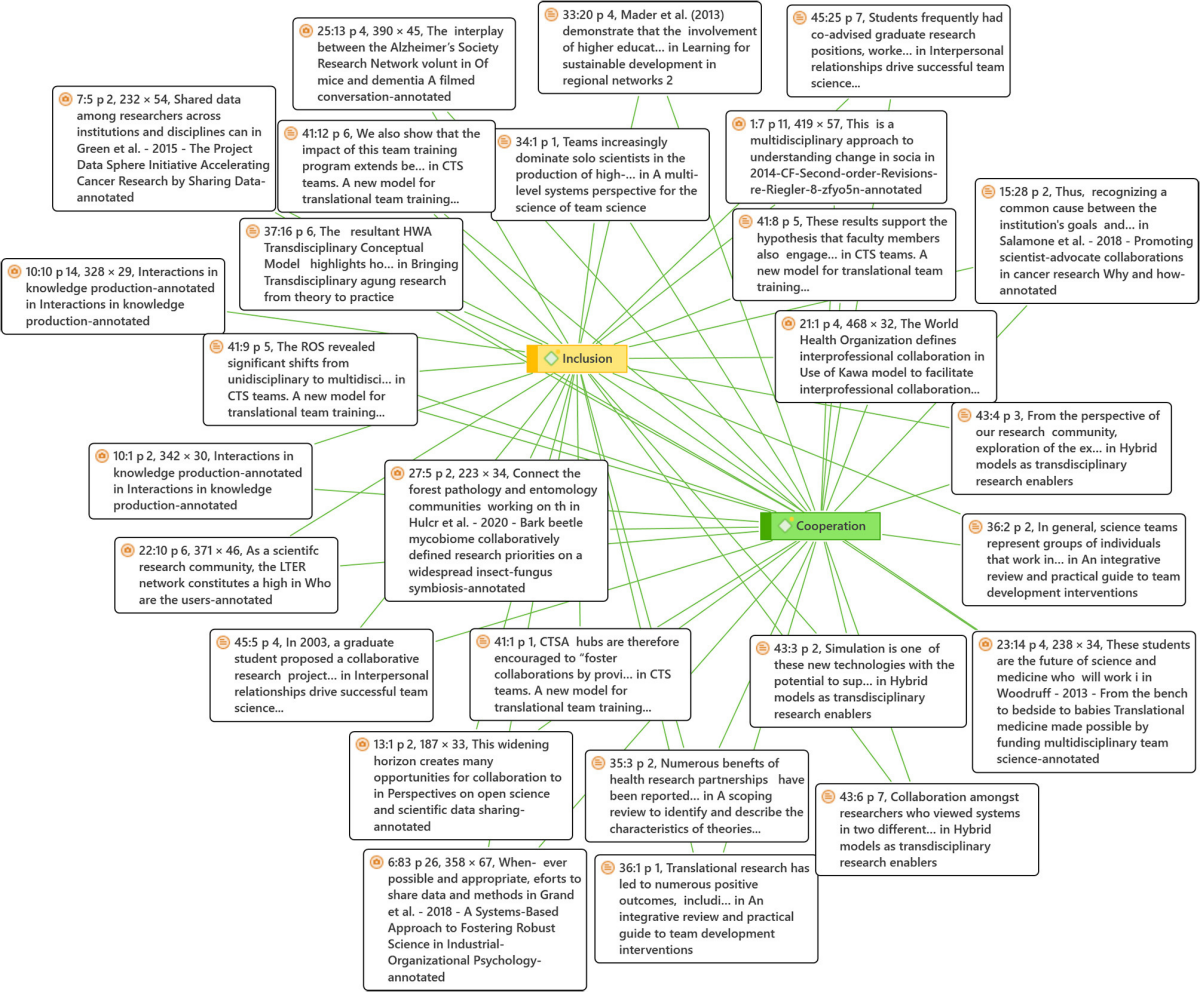
Cooperation and inclusion network. This figure shows the network of co-occurrence of the values of Inclusion and cooperation and some of their citations as examples, generated with Atlas.ti.

**Figure 9:**
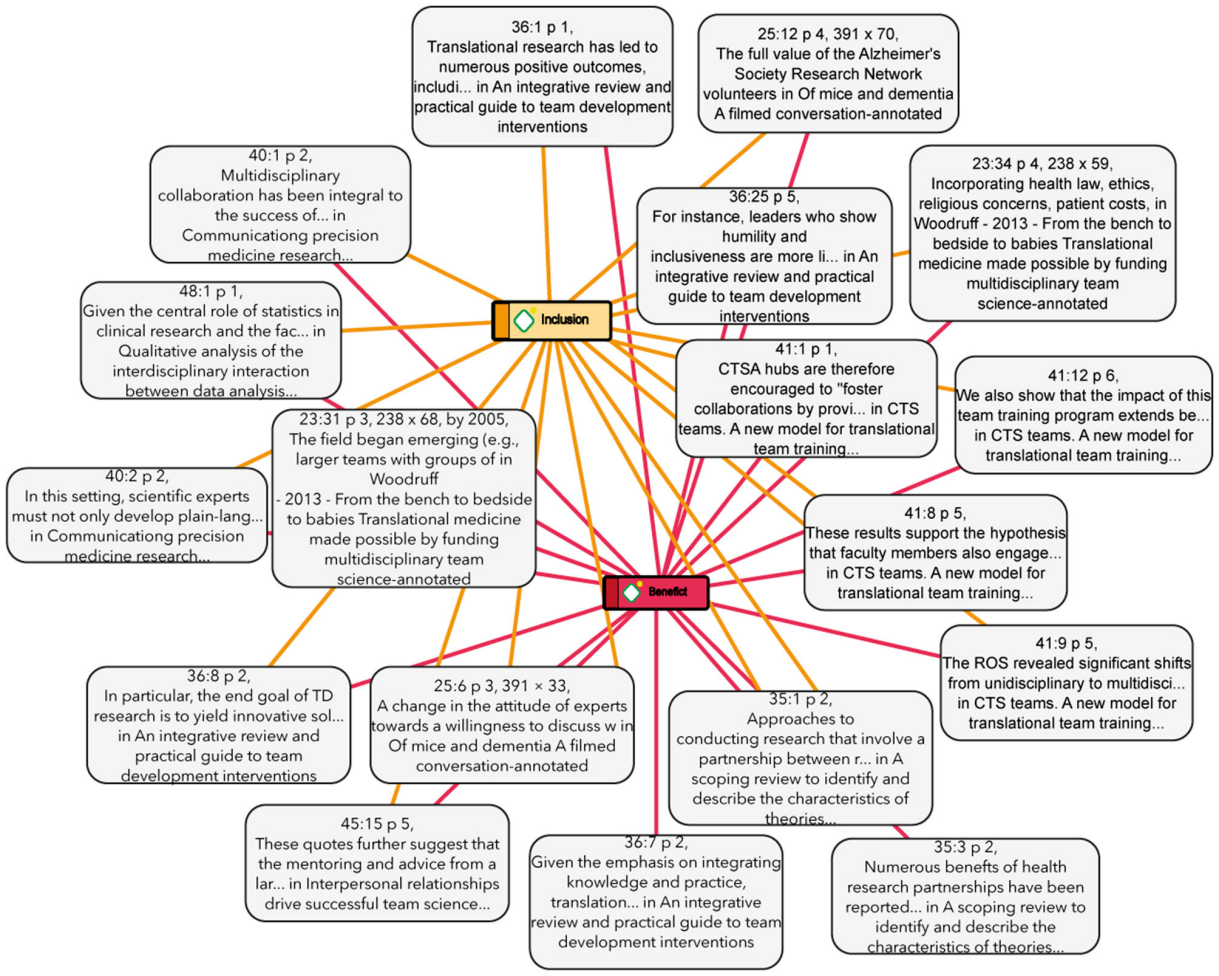
Benefit and inclusion network. The figure presents the network of Co-occurrence between the values Inclusion and Benefit and some citations, generated with Atlas.ti.

**Figure 10:**
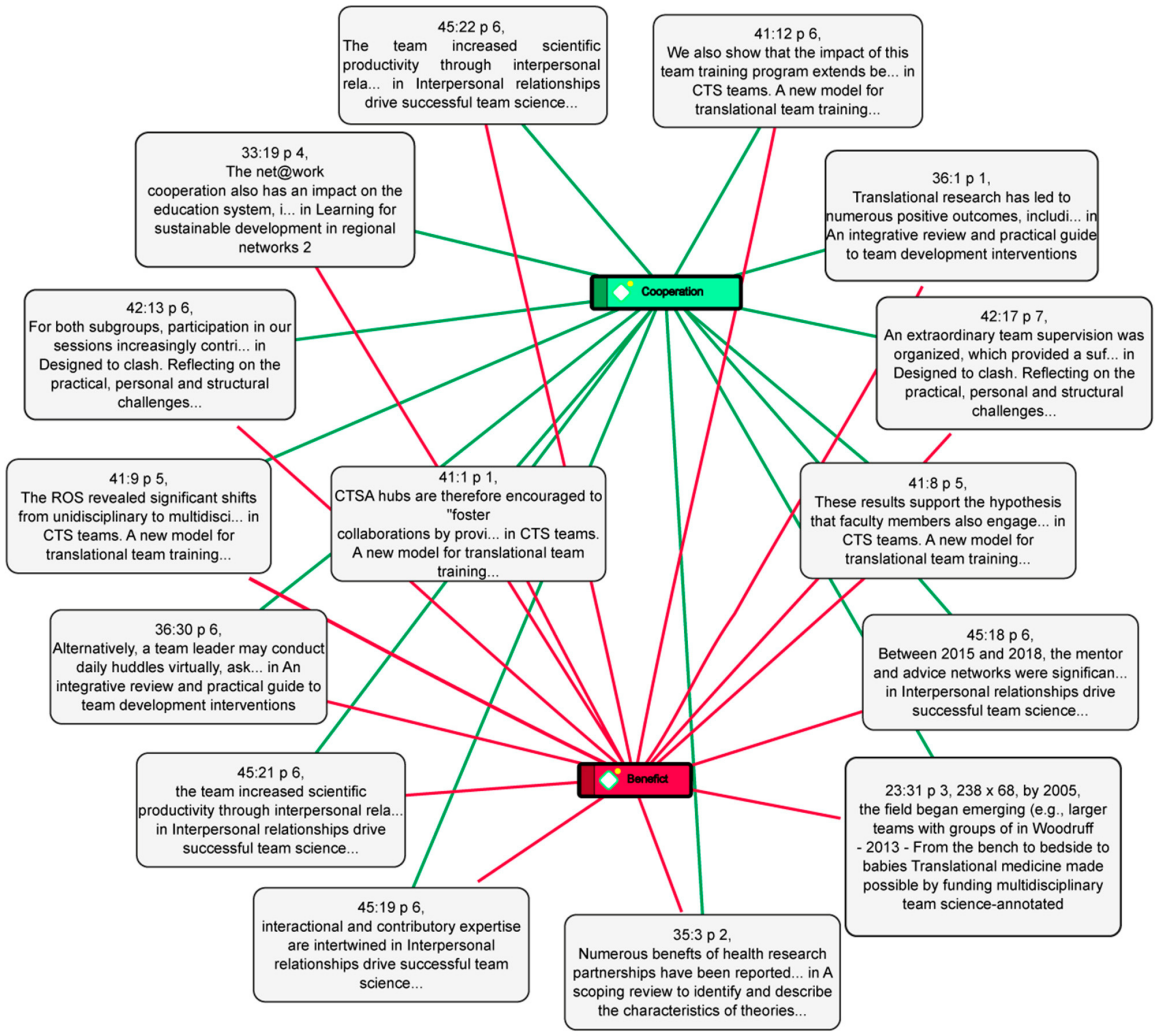
Cooperation and benefit network. This figure presents the co-occurrence network between the values Cooperation and Benefit and some citations as an example, generated with Atlas.ti.

In cross-functional teams, the exchange and translation of knowledge to productive sectors is more effective, as each team member represents a key link in the translational process. This structure encourages basic research areas to be informed by real-world challenges—such as those found in medicine—and to actively seek solutions. As a result, the transfer of biomedical knowledge to clinical practice becomes faster, more efficient, and more direct. In Figures 5–10 we show the key values of this translation process.

#### 3.2.3 The axiological axis as vector of change in research groups

To conduct the analysis, the values were divided into 12 families, as well as, their respective codes: aesthetic, basic or common, ecological, economic, epistemic, legal, military, moral, political, religious, social and technical. The most common value families found in the articles were social values (23%), economic values (20%), and epistemic values (18.5%). Among the least relevant value families according to the investigations were ecological (2.2 %), aesthetic (1.6 %), and religious values (1.4 %). In the following paragraphs the axiological analysis (see Supplementary table of code frequency) and the axiological network are detailed:

It was found that the most frequent values and practices are cooperation, belonging to the family of social values; inclusion, belonging to the family of research practices; benefit, which is part of the family of economic values; adaptation, grouped into the family of epistemic values; and continuous learning, which is part of the good research practice family. These are followed by types of communication, values and practices such as meetings, conversations, understanding, applicability and cohesion. This shows that the five codes with the highest rootedness are part of different families of values or practices that are manifested in the research groups. In this paper, inclusion is understood as an action that invites one or more people to participate in a research activity. It is a scientific practice because it seeks to determine whether this practice is relevant when conducting research within these multidisciplinary groups. This is a key factor in understanding their dynamics, potentially resulting in more fair, rigorous, innovative and effective research (Table 2).

**Table 2:** Most representative values and practices (examples).

**Families**	**Most representative values and practices/selected quotations**
**Social values**	Cooperation: By acting together with others to achieve a common goal Quote: “In cybernetics, when people studied cognition, they brought together scientists from several fields and tried to develop a new understanding of knowledge.” (Umpleby, 2014, p. 13) Solidarity: When you adhere to the task or cause of others Quote: “Informal CoPs are generally voluntary, with rewards often intrinsic and lows levels of sponsorship from senior management. They often evolve naturally and exist for the sole purpose of just sharing knowledge.” (Sung and Parboteeah, 2014, p. 3)
**Economic values**	Benefit: Thinking about a profit Quote: “examine how individual motivations and social capital influence knowledge contribution: individual motivations (reputation, enjoy helping), structural capital (centrality) and cognitive capital (self-rated expertise).” (Sung and Parboteeah, 2014, p. 4) Cost-effectiveness: What is produced generates a sufficient or remunerative income Quote: “making in the U.S. heath care environment, the granularity of the data contained within PDS could also facilitate cost effectiveness and resource utilization analyses for comparison of various treatment strategies.” (Green et al., 2015, p. 6)
**Epistemic values**	Adequation: When it adapts to the needs or conditions of a situation or a thing. Quote: “A flexible approach: using a variety of methods that were employed to engage with the community and to work in partnership.” (Sung and Parboteeah, 2014, p. 4) Clarity: When ideas or reasoning are expressed in a very easy-to-understand way Quote: “authors should accurately describe and represent the capabilities of their methodologies and analyses when proposing inferences from their research.” (Green et al., 2015, p. 15)
**Legal values**	Autonomy: Establish rules of conduct for themselves and in their relationships with others Quote: “the scientific profession continued—and largely still continues—to operate in ways consistent with its decentralized, autonomous, and idiosyncratic heritage.” (Green et al., 2015, p. 6) Transparency: When you act in a clear, evident way and understand it without doubt or ambiguity. Quote: “Second, authors should be precise and transparent when describing the development of research question and the rationale for the methodological/analytical choices used in their research.” (Green et al., 2015, p. 17)
**Religious values**	Charity: An attitude of solidarity with the suffering of others Quote: “Both Ipsos MORI's and Scottish Community Development Centre's models stemmed from a service perspective and did not explicitly recognize engagement as steaming form the community.” (Sung and Parboteeah, 2014, p. 3) Faith: Act with confidence and a good opinion that you have of someone or something Quote: “These assumptions include the belief that the observer should not be included in what is observed and the belief that theories do not affect what is observed. These assumptions would change if second-order science is accepted.” (Umpleby, 2014, p. 9)
**Technical values**	Functionality: Design and organize with ease, utility, and comfort in mind Quote: “the application of knowledge that is supported by robust science to create tools, services, and solutions will be more generalizable reliable.” (Green et al., 2015, p. 25) Innovation: When a product is created or modified, and its introduction into the market Quote: “Innovations or adaptations in both the content and process of delivering interventions often evolve in the context of seeking better solutions to unmet needs faced by particular client groups.” (Sanders and Kirby, 2015, p. 1)
**Aesthetic values**	Creativity: When establishing, founding, or introducing something for the first time Quote: thought about things differently, we would no doubt invent new theories and methods.” (Umpleby, 2014, p. 11) Simplicity: Being natural, spontaneous and the absence of unnecessary artifices Quote: “In contrast, PDS requires submission of only a brief application with information about the background of the individual requesting access and an agreement to terms of use.” (Grand et al., 2018, p. 4)
**Ecological values**	Conservation: When the appropriate resources of an environment are preserved Quote: “We are now in a transition to a new kind of knowledge. It could be called reflexive knowledge or a greater self-awareness as a result of cognitive science and an awareness of our impact on our social and biological environment.” (Umpleby, 2014, p. 2) Balance: When harmony is maintained between diverse things Quote: “However, a balance is needed between meeting service system demands for programs that work with the need to develop credible evidence base to justify the dissemination and scaling up of interventions.” (Sanders and Kirby, 2015, p. 3)
**Research practices**	Cohesion: that encourages meeting or adhesion between the subjects of the group Quote: “Others advise researchers to engage participants as self-determined, establish respectful dialogue, get to know them, and collaboratively define each party's role and the duration of the relationship.” (Mcdonald and Patka, 2012, p. 1) Inclusion: Invite, propose or think of someone within a task or activity Quote: “This widening horizon creates many opportunities for collaboration to scholars from a wide diversity of disciplines.” (Bisol et al., 2014, p. 2)
**Types of communication**	Conversations: Exchange of ideas between two or more people Quote: “This started a dialog about the skills life scientists need in their daily work.” (Stevens et al., 2018, p. 6) Meetings: Formal grouping of several subjects to discuss a matter Quote: “They must regularly attend meetings and become familiar with the process of cancer research and educated in the science of cancer.” (Salamone et al., 2018, p. 3)
**Good practices**	Continuous Learning: Acquire knowledge constantly and regularly Quote: “Training workshops in scientific programming are often offered as one-time courses, but researchers would benefit from more permanent support.” (Stevens et al., 2018, p. 2) Ask for help: Accept someone's cooperation or help. Quote: “We had no problem at all really. There is a lot of respect of each other. We know we couldn't have done this without each other's help. So there is respect.” (Kislov, 2012, p. 202)

This table presents the most representative values resulting from the analysis of the articles, with their definitions and citations as examples.

As explained above, the value system proposed by Echeverría is used as a starting point, given that it is one of the most clearly representative of the complexity of the values involved in technoscience, a complex one due to the emergence of different families of values and values themselves. Another point taken from this proposal is the flexibility within which values are framed, as they are not fixed or immobile values; rather, they are presented as transitive values between different families, as they can be useful for explaining certain actions or attitudes in both frameworks: “There are values, such as freedom, that can be understood in very different ways: as a basic, epistemic value […] political, legal, business, social, etc.” (Echeverría, 2003).

In this same sense, we agree with the position that values have different meanings depending on the context in which they are applied and the conventions they reach in a given space and time (Harman, 2000). For this reason, we believe the value of autonomy is understood as a legal value, since, in the context of medical legislation, it seeks to protect the patient's autonomy from any decision or action that harms their health; or that the value of creativity has a meaning as an aesthetic value, given its relevance to the idea of originality and breaking with the existing.

Figure 5 shows the connections and the degree of centrality of the values and practices with respect to each other, locating the relevance of each of them in relation to the coded papers:

If the family to which these elements belong is indicated, it will be easier to understand the most frequent practices and values among these communities analyzed in the research papers. This is because this data is found based on the total of the rootedness of all the elements of the same family, data that slightly varies when compared with the results of the individual codifications. Figure 6 shows families with the highest amount of rooting or codings:

This way, the most frequent families in the studies of cross-functional research groups are obtained, which indicates the type of practices analyzed or explained by these papers. This reveals that research practices and social, economic and epistemic values, in conjunction with the types of communication, are the most studied concepts. This means that the practices addressed in these studies of the various scientific communities focus on these five topics, and in an underlying way, tacit communication, technical values, good research practices, moral and administrative values, and explicit communication are also addressed. A more detailed analysis of these practices and values will be followed below, but here are only shown those that are most common in current studies.

#### 3.2.4 Generation of the main practical and axiological networks

To identify the most prominent relationships within the practices of the analyzed research groups, we examined the main co-occurrences and interactions between value families and practices in the studies. As each concept is bound to a family, these families were located only with the purpose of later finding the relations among the resulting families. Table 3 shows the major co-occurrence of the values and practices, indicating with different color the family to which each one belongs.

Table 3Relationship between co-occurrences of values, practices, and their families.

**Table:** 

Good practices	Research practices
Economic values	Explicit communication
Epistemic values	Technical values
Moral values	Social values
Tacit communication	Types of communication

**Table:** 

**N of co-occurrences**	**Values and practices**	**Values and practices**
42	Cooperation	Inclusion
19	Benefit	Inclusion
17	Cooperation	Benefit
12	Continuous learning	Cooperation
10	Cooperation	Solidarity
8	Continuous learning	Inclusion
8	Benefit	Meetings
8	Conversations	Meetings
7	Applicability	Cooperation
7	Cooperation	Meetings
6	Altruism	Solidarity
6	Continuous learning	Learnings
6	Cooperation	Respect
6	Adequation	Versatility
5	Adequation	Inclusion
5	Benefit	Clarity
5	Benefit	Training
5	Benefit	Cost-effectiveness
5	Cohesion	Conversations
5	Cohesion	Meetings
5	Inclusion	Interviews
5	Inclusion	Innovation

The table presents the most common co-occurrences between values based on the document analysis, each with a color representing its family for easy identification.

##### 3.2.4.1 Main practices and interaction within the research groups

From the analysis of selected papers, derived from the most relevant values and practices, the practices and the dynamics of the scientific communities or the cross-functional research groups that were found are shown. Those relations among the most representative families were chosen by analyzing their interaction with other families and, with the help of Figure 7, the strength of these relations among the selected families can be clearly seen.

In Figure 7 it can be observed that, given the closeness and thickness of the bonds, the central triangle formed by the families of social, research and economic values are the main practices that were found in the studies, given that they are the ones with the strongest bonds, as well as being related with five or six different families. Meanwhile, the secondary families would be those of types of communication, good research practices, technical values and epistemic values as they are each related to four different families. Finally, the tertiary families are found, those of moral values and tacit and explicit communication, which are related to only one family, respectively. These networks formed with the families represent the three most common practice relations, four secondary practice relations and three tertiary practice relations within the analyzed articles.

These practices are grouped together on five main relations: social values and research practices, research practices and economic values, social values and economic values, research practices and types of communication, and social values and good research practices. Below, how these practices are formed is explained to get a perspective on the structure of the scientific communities studies.

##### 3.2.4.2 Social values and research practices as central axis to the practices of research groups

The relations between these two groups are fundamental to understanding the practices of the research groups. This relation has the most presence among the articles analyzed, given that the co-occurrence among the elements of the families of social values and research practices are the most numerous (42 co-occurrences). This means that these articles place their attention on the research practice in conjunction with social values that play a fundamental role in said practice. The analyzed relation was that of cooperation (social) and inclusion (research), finding that the cooperation value is closely related to the inclusion practice when performing the research task, as represented in Figure 8.

The quotes below show some examples of how inclusion is related to cooperation within the cross-functional research groups:

“Cross-disciplinary projects, including those implemented by the CLAHRCs, can be considered as a variant of boundary practice because participating in this kind of project exposes practitioners to specific tasks going beyond their normal practices and forces them to negotiate their own competencies with the competencies of others” (Kislov, 2012). “Thus, recognizing a common cause between the institution's goals and the patient can logically lead to the establishment of an advocacy committee to collectively facilitate faster clinical advances. By providing appropriate support, resources, and feedback, the institution will help ensure the success of the committee and the achievement of its objectives” (Salamone et al., 2018). “However, by extending the existing breeding oriented networks formally to encompass a broader range of stakeholders, including agronomists, national extension services, agricultural non-government organizations (NGOs), etc., the impact of both national and international crop improvement programs would be multiplied” (Reynolds et al., 2012).

##### 3.2.4.3 The relation between research practices and economic values as another of the fundamental practices of diverse groups

This relation shows how the investigations are exposing the benefits that collaborative practices of inclusion in research had, and specifically the great importance that the relation between (economic) benefit and (research) inclusion has. This relation describes not only the economic benefits of a research practice, but also the benefits regarding the research work and the objectives pursued in it, as facilitators of the research practice to come to answer in a more efficient way the questions posed, etc. Some of the relations are shown in Figure 9.

The most recurrent idea obtained from the analysis was that the inclusion within the scientific communities is beneficial to answer their questions or achieve the objectives posed by the group.

“Approaches to conducting research that involve a partnership between researchers and knowledge users during the research process are now being employed to develop knowledge that is deemed more relevant to knowledge users” (Tittlemier et al., 2022). “For instance, leaders who show humility and inclusiveness are more likely to develop a psychologically safe environment for their team members” (Begerowski et al., 2021). “In this setting, scientific experts must not only develop plain-language explanations for technical concepts but also engage community partners to help them understand salient cultural, material, social, and historical factors that are relevant to the research” (Beans et al., 2022).

##### 3.2.4.4 The relation between social and economics values as another fundamental practice

This relation has as main actors the (economic) benefit and the (social) cooperation. As mentioned in the relation above, the benefit described in the analyzed codes of the documents is not only seen as economic, but also in relation to the group objectives and the tasks to be performed in the research practice. Therefore, the benefits analyzed in the cooperation among the members that belong to diverse scientific communities are reflected both in the aspect of the group efficiency and its impact on the funds or the investment obtained to develop the research conducted. See Figure 10.

The following examples show some kind of benefit that was found in the analyzed studies of certain proposals in the research practice within the research groups:

“The CoP approach has been demonstrated to enhance interprofessional clinical practice (White et al., 2008), facilitate quality improvement, encourage buy-in among participants, promote knowledge transfer (Bentley et al., 2010), and contribute to the development of services spanning the interests of different stakeholders (Lathlean and le May, 2002)” (Kislov, 2012). “The network cooperation also has an impact on the education system, i.e., it supports a transition toward a more open, action-oriented and decentralized system” (Dlouhá et al., 2013).

##### 3.2.4.5 The importance of the relation between the research practice and the types of communication as a relevant practice for these communities

The relations found between these two families are directed toward the ways in which communication has an impact or some kind of influence on the research activity practice. The elements that belong to these relations between these families are the research practices, such as cohesion and inclusion, while in the types of communication are conversations, meetings and interviews. In the analysis, it is highlighted that cohesion and inclusion as research practices are developed more efficiently whenever there are interactions among the members of the research groups, mostly through conversations, meetings and interviews. Some examples of this are shown below:

“Through regular teleconferences, working group members suggested examples to illustrate a range of precision medicine research applications. During this process, the group reached consensus about the scope of precision medicine research and defined next steps to develop case examples” (Beans et al., 2022). “This can be the starting point for a reflection which proceeds from the specific, personal aspects to the construction of more general, abstract concepts, and theories” (Beeker et al., 2021).

In regards to the relation between the meetings and the cohesion achieved in them, the following examples of other analyzed articles are shown:

“Although the exact contents of a team charter may vary from team to team, the initial meeting to form a team charter can also provide a guided discussion format for teams to define their problem space, as is necessary for the development phase” (Begerowski et al., 2021). “For a short period, we used research diaries to document our efforts but had to acknowledge that taking notes separately did not advance a shared understanding. Instead, we started an open and ongoing reflexive process, consisting of special meetings, online discussions, and supervisory sessions for this purpose” (Beeker et al., 2021).

##### 3.2.4.6 The relation between social values and good research practices as frequent practices in research groups

The last relevant relation from the research analysis was between the family of social values and the family of good research practices, where cooperation as social value is closely related with the continuous learning practices within the groups. This suggests that continuous learning is seen as a practice that increases cooperation among the members of these types of groups, an idea that is proved by the following examples:

“In a socially situated view of learning, individuals continuously combine and modify knowledge through their everyday operations and interactions between each other” (Kislov, 2012). “The team started a learning collaborative led by three members who further explored this question and developed a de-identified survey which was completed by all HWA members” (Sargent et al., 2022).

This way, the main practices present in these current communities or research groups are described, based on the studies. These practices show the relevance of social and economic values, good research practices and the types of communication established among these group members. These relations are found to be most frequently represented in the study offered by the articles, but it does not indicate that they are the most preferable or those that work best to achieve a good interaction and communication among these group professionals.

In order to find the dynamics that expose the most recommendable practices for these diverse groups, it is necessary to conduct another type of analysis, which studies the advantages and disadvantages of the practices that are presented as desirable, and, if it is possible, to conduct or review an empirical study that serves as contrast to some proposal in this direction. In the future, a research that focuses on these problems will be designed, to try to propose a model or framework of recommended practices in the scientific work that exists in scientific communities or cross-functional research groups.

## 4 Phase three—Discussion

In the systematic review, it was found that the relevance of theoretical analyses is greater than that of empirical studies, specifically two thirds of the articles analyzed belong to theoretical studies, while the remaining third belongs to empirical studies. As shown in the results section, this indicates that most studies rely on conceptual analyses or systematic reviews to examine the structure and configuration of research groups (Sanders and Kirby, 2015; Weiskopf, 2020; Beans et al., 2022). This indicates that elements characteristic of empirical studies, such as relation, interactions, types of communication or socialization among the members have not been addressed in a comprehensive manner. This leaves a wide margin of work for future research and a field in which research must be continued if what is wanted is to propose current models of practices or dynamics of the scientific or cross-functional communities. It should be highlighted that the difficulty that conducting empirical studies represents is greater than that of a theoretical analysis, given that the first one requires more study time for the selection of the group to be investigated, for data collection or data analysis and interpretation, etc. That is why the empirical study faces several logistical problems, transfers or voyages for observation or implementation of data collection tools. On the other hand, the second type of study does not require the investment, for the most part, of so many material or human resources, given that it is possible to use recorded data, archives, databases or results of publications or articles, and, based on these, conduct a theoretical analysis that would suppose fewer problems than an intervention. Therefore, the value of empirical studies resides in that required investment of material and human resource, and overall, in the fact that the results of these studies are directly related to interventions—whether through observation, interviews or surveys and workshops—that describe social interactions in specific contexts, since most of the interventions that are conducted are case studies. What can also be made in this type of studies, and it is of great value, is to find the most common contexts in which this type of group interacts. This way, frameworks that explain better their practices and reflect a higher percentage of these research societies are constructed.

Given that the studies found are mostly a part of a theoretical work, it should be highlighted that the minority of these investigations—the majority of the empirical studies—focus on presenting a proposal or a model that tries to improve the practices of these diverse research groups. This is done through the implementation of courses or workshops that have the objective of teaching their participants alternative forms of interaction, communication or practices within these groups (Roa-Atkinson and Velho, 2005; Beeker et al., 2021; McCormack and Levites Strekalova, 2021). Although descriptive studies expose and show the type of practices prevailing in the analyzed groups, it will be more recommended to put into practice a proposal already worked theoretically and/or empirically in order to know its advantages and disadvantages. This applies whenever there is valuable evidence for the reproduction or omission of practices that improve their socialization and communication processes, since this would be one step closer to proposing better practices for these diverse groups in specific contexts.

It should be highlighted that in most of the articles found, the tacit elements of information or knowledge exchange are presented on a regular basis. These elements were the most relevant when making the codification of the articles, which remarks the importance given to this type of knowledge acquisition in the analyses conducted in research groups studied in the articles. This is because these practices, together with the explicit ones, are vital for the exchange of information that is not necessarily taken into account, given that they are not conducted by formal tools, such as documents or manuals (Pahl-Wostl, 2007; Grand et al., 2018; Beeker et al., 2021). Attending to key elements, such as previous experiences or learnings, indicates that the knowledge shared among the members of these research groups keeps a direct interaction among them, because, as described in the following point, contact or personal interaction is a practice that remains active despite having technological tools (ICT) at their disposal. From this dynamic of science, conflicts or disputes between scientific groups or communities are expected when a paradigm or a theory is challenged. This issue, moreover, affects any type of group and is central, since these are academic disputes directed at specific theoretical points, and a purely objective discussion should prevail (Scotognella, 2021). However, as suggested in this work, the values and practices that arise from them are charged with political, economic, social, and even religious values.

One of the key features of cross-functional groups is the learning processes and practices that emerge throughout integrative research. In such interdisciplinary settings, group members contribute diverse knowledge, skills, methodologies, and approaches to solving the target problem. These varied perspectives alternate in prominence, as individuals act both as experts in their own domains and as non-experts in others. This dynamic fosters a collaborative learning and innovative environment where mutual knowledge exchange is not only encouraged but essential. The constant interplay between different areas of expertise enables members to learn from each other and develop a shared understanding and common epistemic language. Through this teaching-learning process, the collective knowledge can shape the foundational capabilities of the research group. Additionally, early-career scientists who engage with cross-functional research from the beginning of their training benefit significantly. Exposure to cross-functional collaboration helps them understand how seasoned experts approach problems, structure research questions, and navigate solution pathways—providing valuable insight into both the content and the process of scientific inquiry.

Since it is interesting to find that, despite the fact that most of the articles are < 5 years old, most of the information exchange practices described in the studied groups take place in face-to-face meetings. The exchange of information or ideas in these meetings is given through conversations or interviews among the members of these groups. As already mentioned, traditional means are mostly used to interact with tacit elements, while currently digital tools—that are common and are available to most of them—represent a small percentage (Woodruff, 2013; Beeker et al., 2021; Begerowski et al., 2021). This can be explained because the members of the analyzed groups are not yet fully familiar with these digital resources and prefer to have information and observations exchanges through traditional means, resulting in the tacit type of knowledge being more common among their members.

Given that the great majority of the analyzed papers pay attention to academic motives as the main or only objective of communication among the members of the research groups, it is necessary to work on and pay more attention to other motives for information exchange, such as social, cultural or personal motives. Among the results obtained, it is shown that the studies conducted by Sung and Parboteeah (2014) or Beeker et al. (2021) have a great impact when it comes to providing information on the type of personal and social relationships that are presented in these types of groups. This is because, as they are groups that in many cases are long-lasting, they form long relationships that can go beyond academic boundaries and strengthen values of friendship and companionship, which would lead to consider that the communication of this type of information can influence the group's practices. In the case of the authors mentioned above, this relationship has a positive influence by improving the cohesion and integration of group members.

The conducted analysis of the three most common practices found observe a strong relation among social, research and economic values. This means that social values, such as cooperation and solidarity; together with research values, such as inclusion and cohesion; and economic values, such as benefit and profitability, were the most representative when studying the practices of the diverse research groups found in the analysis of the investigations. This information allows us to understand that the most common practices of these groups are related to values such as the social organization of the group, supporting a reach of good levels of cooperation and solidarity within the group in order to efficiently achieve its objectives. Research values are also relevant to these groups, since it is precisely the research activity what unites and motivates them to achieve their joint goals. That is why values such as inclusion and cohesion are the most common among their practices, given that, with them, the objective is a joint collaboration that accepts different perspectives and opinions on the problems being worked on. Finally, the economic values of benefit and profitability are frequently presented in the study, suggesting that the benefits obtained from the research activity of the group are important for its members—whether they are personal or group benefits at academic level—and their practices, in addition to the fact that economic benefits will be sought if the results of the research yield products with the possibility of being introduced in the market.

One of the most relevant findings in this investigation is the identification of the network of values in the research practice that constitute the organizational axiological framework of decision making in which the values are dynamic. These traits allow values to be flexible and become relevant in different scenarios, Table 2 and Figure 5.

**Figure 11:**
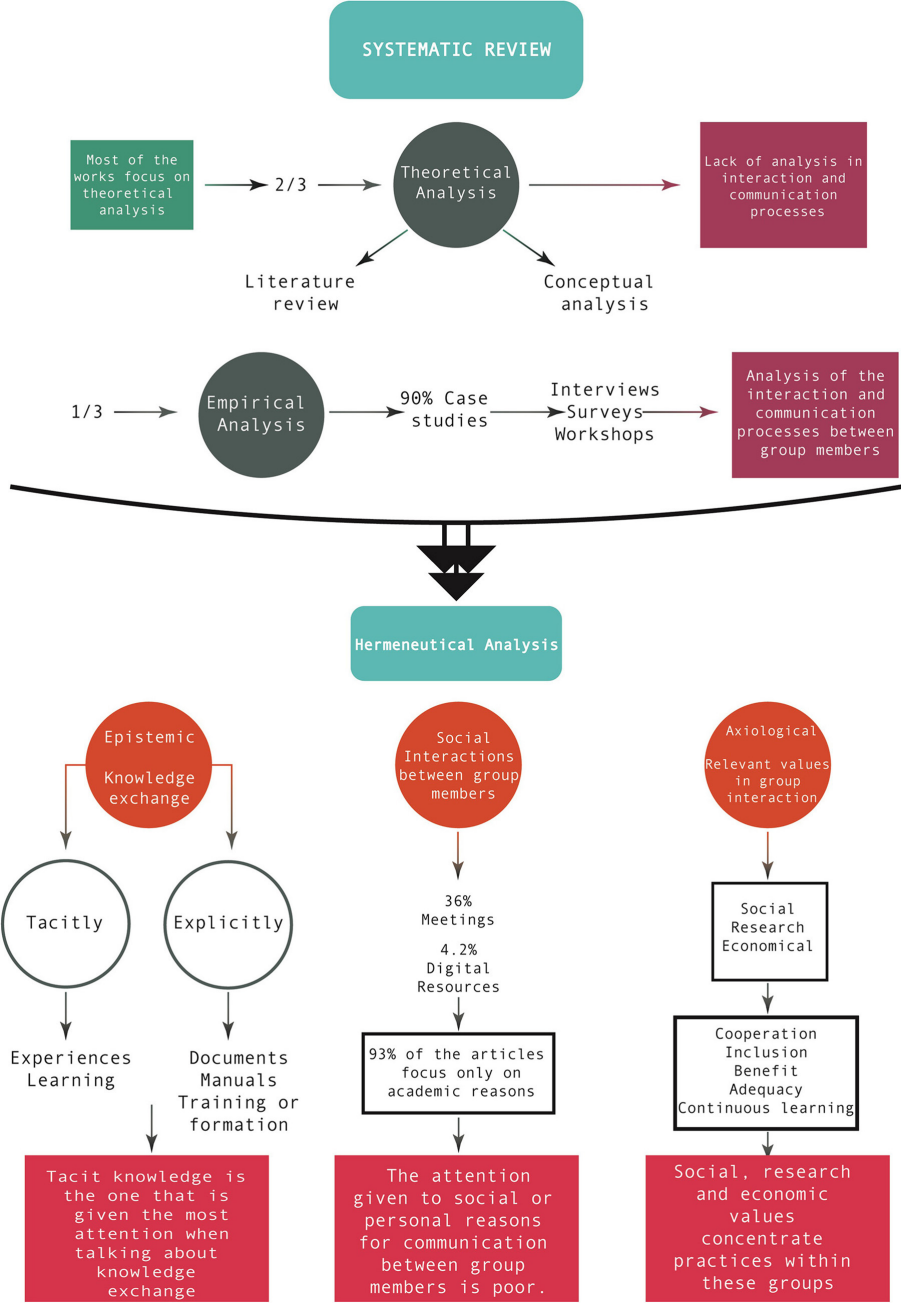
Highlighted points analysis. The figure presents a summary of the main research findings, both from the systematic review and the meta-analysis.

This above indicates the type of study and the topics of interest that directed these researches. Therefore, it would be interesting that subsequent studies of diverse research groups address the practices that take precedence in them from other perspectives, so that practices centered on social values that strengthen the processes of interaction communication or interrelations among the members of these groups can be proposed. This way, a wider analysis of this type of groups would be formed, resulting in proposals or models of more comprehensive practices. The previous highlighted points in the analysis are presented in Figure 11.

## 5 Conclusion

This study highlights the importance of practices in diverse research groups. Through a systematic review, we identified various models and frameworks for group dynamics. As already shown, these proposals are made in diverse ways, some from a theoretical perspective, while others from an empirical perspective. Empirical studies are more effective in identifying representative practices within group interactions. This does not mean that theoretical studies are not relevant for the study of practices, but rather that, as said above, these studies are interesting because they attempt to find the points in common among the different proposals studied from a more general perspective, and, this way, create general models that attempt to answer most of the contexts in which these groups operate. This is why, if one wants to analyze diverse research groups in their different contexts, it is proposed that this type of studies choose to conduct an empirical study, which exposes specific dynamics in different contexts already tested in the field.

It was also highlighted that the social or personal elements of interaction and communication among the members of these groups are important when we want to describe their practices and dynamics in a comprehensive manner. This is because these elements actively intervene in the integration, cohesion and cooperation of the group. Academic dynamics reveal the formal activities and professional practices of group members, where professional practices that each of the members have been assimilating and learning in their professional training are outlined. However, these academic dynamics represent only one of the factors involved in the construction of the social dynamics of the group, so that by paying attention to other non-formal factors, a more complete explanation of their dynamics can be reached.

From the practices described in these works, it was observed that social, research, and economic values emerged as the most prominent. According to the found results, the work that requires form a construction of a cooperative and supportive environment within a group is fundamental to achieve the expected objective. Likewise, attitudes of inclusion and cohesion among group members were found to be essential for effective research. Lastly, it was observed that economic values are relevant in the dynamics of the groups, both personal or group benefits, as well as those that intend to find market values in the results of their activity.

It is important to highlight that these findings reflect a context in which contemporary science and knowledge production are shaped by political, economic, social, and cultural factors. For several decades, this type of science context has run the path that scientific activities walk. Therefore, it is also important to pay attention to these practices to achieve a balance among the factors involved in these activities, so the scale does not only tip for the factor with economic and business purposes.

## Data Availability

The original contributions presented in the study are included in the article/Supplementary material, further inquiries can be directed to the corresponding author/s.

## References

[B1] Andersen H. (2016). Collaboration, interdisciplinarity, and the epistemology of contemporary science. Stud. Hist. Philos. Sci. Part A 56, 1–10. 10.1016/j.shpsa.2015.10.006 27083079

[B2] Ankeny R. A. Leonelli S. (2016). Repertoires: a post-Kuhnian perspective on scientific change and collaborative research. Stud. Hist. Philos. Sci. Part A 60, 18–28. 10.1016/j.shpsa.2016.08.003 27938718

[B3] Bainbridge L. Nasmith L. Orchard C. Wood V. (2010). Competencies for interprofessional collaboration. J. Phys. Ther. Educ. 24, 6–11. 10.1097/00001416-201010000-00003

[B4] Báscolo E. Yavich N. Sánchez de León A. (2006). El proceso de interacción investigadores y tomadores de decisiones: un estudio de caso. TT - [Interaction between researchers and decision-makers: a case study]. Cad Saude Publica 22(Suppl), S47–56. 10.1590/S0102-311X2006001300014 17086337

[B5] Beans J. A. Trinidad S. B. Blacksher E. Hiratsuka V. Y. Spicer P. Woodahl E. L. . (2022). Communicating precision medicine research: multidisciplinary teams and diverse communities. Public Health Genom. 25, 155–163. 10.1159/000525684 35998578 PMC9947193

[B6] Beeker T. Glück R. K. Ziegenhagen J. Göppert L. Jänchen P. Krispin H. . (2021). Designed to clash? Reflecting on the practical, personal, and structural challenges of collaborative research in psychiatry. Front. Psychiatry 12:701312. 10.3389/fpsyt.2021.701312 34305686 PMC8292740

[B7] Begerowski S. R. Traylor A. M. Shuffler M. L. Salas E. (2021). An integrative review and practical guide to team development interventions for translational science teams: one size does not fit all. J. Clin. Transl. Sci. 5:e198. 10.1017/cts.2021.832 34888067 PMC8634301

[B8] Bentley C. Browman G. P. Poole B. (2010). Conceptual and practical challenges for implementing the communities of practice model on a national scale—a Canadian cancer control initiative. BMC Health Serv. Res. 1:3. 20051125 10.1186/1472-6963-10-3 PMC2820037

[B9] Bisol G. D. Anagnostou P. Capocasa M. Bencivelli S. Cerroni A. Contreras J. . (2014). Perspectives on open science and scientific data sharing: an interdisciplinary workshop. J. Anthropol. Sci. 92, 179–200. 10.4436/JASS.92006 25020017

[B10] Börner K. Contractor N. Falk-Krzesinski H. Fiore S. Hall K. Keyton J. . (2010). A multi-level systems perspective for the science of team science. Sci. Transl. Med. 2. 1–9. 10.1126/scitranslmed.3001399 20844283 PMC3527819

[B11] Braun T. Schubert A. (2003). A quantitative view on the coming of age of interdisciplinarity in the sciences 1980-1999. Scientometrics 58, 183–189. 10.1023/A:1025439910278

[B12] Dlouhá J. Barton A. Huisingh D. Adomssent M. (2013). Learning for sustainable development in regional networks. J. Clean. Prod. 49, 1–4. 10.1016/j.jclepro.2013.01.041

[B13] Echeverría J. (2003). La Revolución Tecnocientí*fica*. Madrid: Fondo de Cultura Económica.

[B14] Erden Z. Klang D. Sydler R. von Krogh G. (2015). “How can we signal the value of our knowledge?” Knowledge-based reputation and its impact on firm performance in science-based industries. Long Range Plann. 48, 252–264. 10.1016/j.lrp.2014.07.003

[B15] Fertman C. I. Dotson S. Mazzocco G. O. Reitz S. M. (2005). Challenges of preparing allied health professionals for interdisciplinary practice in rural areas. J. Allied Health 34, 163–168. 16252679

[B16] Grand J. A. Rogelberg S. G. Allen T. D. Landis R. S. Reynolds D. H. Scott J. C. . (2018). A systems-based approach to fostering robust science in industrial-organizational psychology. Ind. Organ. Psychol. 11, 4–42. 10.1017/iop.2017.55

[B17] Green A. K. Reeder-Hayes K. E. Corty R. W. Basch E. Milowsky M. I. Dusetzina S. B. . (2015). The project data sphere initiative: accelerating cancer research by sharing data. Oncologist 20, 464–e20. 10.1634/theoncologist.2014-0431 25876994 PMC4425388

[B18] Harman G. (2000). Explaining Value: And Other Essays in Moral Philosophy. Oxford: Oxford University Press UK. 10.1093/0198238045.001.0001

[B19] Hoekstra F. Mrklas K. J. Sibley K. M. Nguyen T. Vis-Dunbar M. Neilson C. J. . (2018). A review protocol on research partnerships: a coordinated multicenter team approach. Syst. Rev. 7, 1–14. 10.1186/s13643-018-0879-2 30497527 PMC6267881

[B20] Hulcr J. Barnes I. De Beer Z. W. Duong T. A. Gazis R. Johnson A. J. . (2020). Bark beetle mycobiome: collaboratively defined research priorities on a widespread insect-fungus symbiosis. Symbiosis 81, 101–113. 10.1007/s13199-020-00686-9

[B21] Juárez-Villegas L. E. Altamirano-Bustamante M. M. Zapata-Tarrés M. M. (2021). Decision-making at end-of-life for children with cancer: a systematic review and meta-bioethical analysis. Front. Oncol. 11:739092. 10.3389/fonc.2021.739092 34722289 PMC8554195

[B22] Kislov R. (2012). Multiprofessional communities of practice in a large-scale healthcare knowledge mobilisation initiative: A qualitative case study of boundary, identity and knowledge sharing. Manchester: The University of Manchester, 1–274.

[B23] Lape J. E. Lukose A. Ritter D. R. M. Scaife B. D. (2018). Use of the Kawa model to facilitate interprofessional collaboration: a pilot study. Internet J. Allied Health Sci. Prac. 17:3. 10.46743/1540-580X/2019.1780

[B24] Lathlean J. le May A. (2002). Communities of practice: an opportunity for interagency working. J. Clin. Nurs. 3, 394–398. 10.1046/j.1365-2702.2002.00630.x 12010537

[B25] Lienert J. Linkov I. (2019). Editorial featured papers on environmental decisions. EURO J. Decision Processes 7, 151–157. 10.1007/s40070-019-00108-2

[B26] Love H. B. Cross J. E. Fosdick B. Crooks K. R. VandeWoude S. Fisher E. R. (2021). Interpersonal relationships drive successful team science: an exemplary case-based study. Humanit. Soc. Sci. Commun. 8, 1–10. 10.1057/s41599-021-00789-838617731

[B27] McCormack W. T. Levites Strekalova Y. A. (2021). CTS teams: a new model for translational team training and team science intervention. J. Clin. Transl. Sci. 5, 1–9. 10.1017/cts.2021.854 34849258 PMC8596063

[B28] Mcdonald K. Patka M. (2012). “There is no black or white”: scientific community views on ethics in intellectual and developmental disability research. J. Policy Prac. Intellectual Disabil. 9, 206–214. 10.1111/j.1741-1130.2012.00348.x29292211

[B29] Méndez Jiménez J. (2014). “Etnografía de un grupo transfuncional en ética clínica,” in Valores y Virtudes en Medicina, eds. Altamirano M. M. Olivé L. Altamirano N. Garduño J. (México D.F.: Corinter), 171–185.

[B30] Millerand F. Baker K. S. (2010). Who are the users? Who are the developers? Webs of users and developers in the development process of a technical standard. Information Syst. J. 20, 137–161. 10.1111/j.1365-2575.2009.00338.x

[B31] Monroy-Fraustro D. Maldonado-Castellanos I. Aboites-Molina M. Rodríguez S. Sueiras P. Altamirano-Bustamante N. F. . (2021). Bibliotherapy as a non-pharmaceutical intervention to enhance mental health in response to the COVID-19 pandemic: a mixed-methods systematic review and bioethical meta-analysis. Front. Public Health 9:629872. 10.3389/fpubh.2021.629872 33796496 PMC8007779

[B32] Olivé Morett L. (2011). Tipos de conocimientos y prácticas epistémicas. Estudios filosóficos 60(173), 9–26 60.

[B33] Pahl-Wostl C. (2007). The implications of complexity for integrated resources management. Environ. Model. Softw. 22, 561–569. 10.1016/j.envsoft.2005.12.024

[B34] Polanyi M. (1966). The Tacit Dimension, 1st Edn. New York, NY: Doubleday & Anchor.

[B35] Reynolds M. P. Hellin J. Govaerts B. Kosina P. Sonder K. Hobbs P. . (2012). Global crop improvement networks to bridge technology gaps. J. Exp. Bot. 63, 1–12. 10.1093/jxb/err241 21926090

[B36] Ricoeur P. (1991). From Text to Action. Illinois: Northwestern University Press.

[B37] Roa-Atkinson A. Velho L. (2005). Interactions in knowledge production: a comparative case study of immunology research groups in Colombia and Brazil. Aslib Proc. 57, 200–216. 10.1108/00012530510599172

[B38] Salamone J. M. Lucas W. Brundage S. B. Holloway J. N. Stahl S. M. Carbine N. E. . (2018). Promoting scientist-advocate collaborations in cancer research: why and how. Cancer Res. 78, 5723–5728. 10.1158/0008-5472.CAN-18-1600 30120210 PMC6548189

[B39] Sanders M. R. Kirby J. N. (2015). Surviving or thriving: quality assurance mechanisms to promote innovation in the development of evidence-based parenting interventions. Prev. Sci. 16, 421–431. 10.1007/s11121-014-0475-1 24610566

[B40] Sargent L. Slattum P. Brooks M. Gendron T. MacKiewicz M. Diallo A. . (2022). Bringing transdisciplinary aging research from theory to practice. Gerontologist 62, 159–168. 10.1093/geront/gnaa214 33349850 PMC8827331

[B41] Scotognella F. (2021). Scientist as parrhesiastes. Eur. Sci. J. 17.

[B42] Serrano-Zamago A. B. Altamirano-Bustamante M. M. (2021). Appealing to tacit knowledge and axiology to enhance medical practice in the COVID-19 pandemic: a systematic review and hermeneutic bioethical analysis. Front. Public Health 9:686773. 10.3389/fpubh.2021.686773 34956997 PMC8692268

[B43] Snyder H. M. Shineman D. W. Friedman L. G. Hendrix J. A. Khachaturian A. Le Guillou I. . (2016). Guidelines to improve animal study design and reproducibility for Alzheimer's disease and related dementias: for funders and researchers. Alzheimer. Dementia 12, 1177–1185. 10.1016/j.jalz.2016.07.001 27836053

[B44] Stevens S. L. R. Kuzak M. Martinez C. Moser A. Bleeker P. Galland M. (2018). Building a local community of practice in scientific programming for life scientists. PLoS Biol. 16:e2005561. 10.1371/journal.pbio.2005561 30485260 PMC6287879

[B45] Sung H.-Y. Parboteeah P. (2014). “A community engagement theory perspective on communities of practice for knowledge sharing,” in 11th International Conference on Intellectual Capital, Knowledge Management & Organisational Learning (Sydney, NSW).

[B46] Tittlemier B. J. Cooper J. Steliga D. Woodgate R. L. Sibley K. M. (2022). A scoping review to identify and describe the characteristics of theories, models and frameworks of health research partnerships. Health Res. Policy Syst. 20. 10.1186/s12961-022-00877-4 35717196 PMC9206347

[B47] Tolk A. Harper A. Mustafee N. (2021). Hybrid models as transdisciplinary research enablers. Eur. J. Oper. Res. 291, 1075–1090. 10.1016/j.ejor.2020.10.010 33078041 PMC7558239

[B48] Umpleby S. A. (2014). Second-order Science: Logic, Strategies, Methods. Available at: http://www.univie.ac.at/constructivism/journal/10/1 (Accessed November 20, 2022).

[B49] Vedrenne-Gutiérrez F. Altamirano-Bustamante M. M. Monroy-Fraustro D. de Hoyos Bermea A. López-Suero C. (2021). Context and implications document for: teaching sciences and mathematics—A challenge for higher education institutions: a systematic review. Rev. Educ. 9, 722–724. 10.1002/rev3.3260

[B50] Weiskopf D. A. (2020). Representing and coordinating ethnobiological knowledge. Stud. Hist. Philos. Sci. Part C 84:101328. 10.1016/j.shpsc.2020.101328 32771278

[B51] White D. Suter E. Parboosingh I. Taylor E. (2008). Communities of practice: Creating opportunities to enhance quality of care and safe practices. Healthc. Q. 11, 80–84. 10.12927/hcq.2008.19654 18382166

[B52] Woodruff T. K. (2013). From the bench to bedside to babies: translational medicine made possible by funding multidisciplinary team science. J. Assist. Reprod. Genet. 30, 1249–1253. 10.1007/s10815-013-0082-2 23975192 PMC3824858

[B53] Zammar G. R. Shah J. Ferreira A. P. B. Cofiel L. Lyles K. W. Pietrobon R. (2010). Qualitative analysis of the interdisciplinary interaction between data analysis specialists and novice clinical researchers. PLoS ONE 5:e9400. 10.1371/journal.pone.0009400 20195374 PMC2827555

