# Supplementary material for: Axiology and dynamics of contemporary research groups: a systematic review and hermeneutic meta-analysis of knowledge, values, and social elements
Source: Front Res Metr Anal. 2025 Aug 7;10:1525587. doi: 10.3389/frma.2025.1525587 (PMC12369186; doi:10.3389/frma.2025.1525587)
Supplement: Supplementary file 1 [file Table_1.docx]

| **ARTICLE TITLE** | **QUALITY CRITERIA** | | | | | **ASSESSMENT** |
| --- | --- | --- | --- | --- | --- | --- |
|  | OBJECTIVES | RESEARCH QUESTION | METHODOLOGY | RELEVANT TERMS | RESULTS |  |
| "How can We Signal the Value of Our Knowledge?" Knowledge-based Reputation and its Impact on Firm Performance in Science-based Industries | 20 | 20 | 10 | 20 | 20 | 90 |
| "There is No Black or White": Scientific Community Views on Ethics in Intellectual and Developmental Disability Research | 20 | 10 | 20 | 20 | 20 | 90 |
| A community engagement theory perspective on communities of practice for knowledge sharing | 20 | 20 | 15 | 20 | 15 | 90 |
| A Multi-Level Systems Perspective for the Science of Team Science | 20 | 20 | 20 | 15 | 20 | 95 |
| A scoping review to identify and describe the characteristics of theories, models and frameworks of health research partnerships | 20 | 20 | 20 | 15 | 20 | 95 |
| A Systems-Based Approach to Fostering Robust Science in Industrial-Organizational Psychology | 20 | 5 | 10 | 20 | 20 | 75 |
| An integrative review and practical guide to team development interventions for translational science teams: One size does not fit all | 20 | 20 | 20 | 20 | 20 | 100 |
| Bark beetle mycobiome: collaboratively defined research priorities on a widespread insect-fungus symbiosis | 15 | 10 | 10 | 20 | 15 | 70 |
| Bringing Transdisciplinary Aging Research From Theory to Practice | 15 | 15 | 10 | 20 | 15 | 75 |
| Building a local community of practice in scientific programming for life scientists | 20 | 20 | 15 | 20 | 20 | 95 |
| Challenges of preparing allied health professionals for interdisciplinary practice in rural areas | 20 | 15 | 15 | 20 | 15 | 85 |
| Communicating Precision Medicine Research: Multidisciplinary Teams and Diverse Communities | 20 | 20 | 20 | 20 | 20 | 100 |
| CTS teams: a new model for translational team training and team science intervention | 20 | 20 | 15 | 15 | 20 | 90 |
| Designed to Clash? Reflecting on the Practical, Personal, and Structural Challenges of Collaborative Research in Psychiatry | 20 | 20 | 10 | 20 | 20 | 90 |
| Editorial featured papers on environmental decisions | 10 | 5 | 5 | 5 | 5 | 30 |
| El proceso de interacción investigadores y tomadores de decisiones: un estudio de caso. TT - [Interaction between researchers and decision-makers: a case study]. | 20 | 15 | 20 | 15 | 20 | 90 |
| From the bench to bedside to babies: translational medicine made possible by funding multidisciplinary team science. | 20 | 12 | 12 | 20 | 15 | 79 |
| Guidelines to improve animal study design and reproducibility for Alzheimer&#039;s disease and related dementias: For funders and researchers. | 20 | 12 | 18 | 20 | 15 | 85 |
| Hybrid models as transdisciplinary research enablers | 20 | 20 | 15 | 20 | 20 | 95 |
| Interactions in knowledge production: A comparative case study of immunology research groups in Colombia and Brazil | 20 | 15 | 18 | 12 | 20 | 85 |
| Interpersonal relationships drive successful team science: an exemplary case-based study | 20 | 20 | 20 | 20 | 20 | 100 |
| Learning for sustainable development in regional networks | 20 | 15 | 10 | 10 | 10 | 65 |
| Perspectives on open science and scientific data sharing: An interdisciplinary workshop | 20 | 15 | 10 | 20 | 20 | 85 |
| Promoting scientist-advocate collaborations in cancer research: Why and how | 20 | 12 | 15 | 15 | 20 | 82 |
| Qualitative Analysis of the Interdisciplinary Interaction between Data Analysis Specialists and Novice Clinical Researchers | 20 | 12 | 20 | 15 | 15 | 82 |
| Representing and coordinating ethnobiological knowledge | 20 | 13 | 15 | 20 | 15 | 83 |
| Second-Order Science: Logic, Strategies, Methods | 20 | 10 | 15 | 20 | 15 | 80 |
| Surviving or Thriving: Quality Assurance Mechanisms to Promote Innovation in the Development of Evidence-Based Parenting Interventions | 20 | 12 | 15 | 15 | 15 | 77 |
| The implications of complexity for integrated resources management | 20 | 12 | 12 | 15 | 18 | 77 |
| The Project Data Sphere Initiative: Accelerating Cancer Research by Sharing Data | 20 | 15 | 15 | 20 | 15 | 85 |
| Use of the Kawa Model to Facilitate Interprofessional Collaboration: A Pilot Study | 20 | 20 | 10 | 15 | 15 | 80 |
| Who are the users? Who are the developers? Webs of users and developers in the development process of a technical standard | 20 | 15 | 20 | 15 | 20 | 90 |
